# HSV-2 as a biomarker of HIV epidemic potential in female sex workers: meta-analysis, global epidemiology and implications

**DOI:** 10.1038/s41598-020-76380-z

**Published:** 2020-11-09

**Authors:** Hiam Chemaitelly, Helen A. Weiss, Laith J. Abu-Raddad

**Affiliations:** 1 Infectious Disease Epidemiology Group, Weill Cornell Medicine-Qatar, Cornell University, Qatar Foundation – Education City, P.O. Box 24144, Doha, Qatar; 2 World Health Organization Collaborating Centre for Disease Epidemiology Analytics On HIV/AIDS, Sexually Transmitted Infections, and Viral Hepatitis, Weill Cornell Medicine-Qatar, Cornell University, Qatar Foundation – Education City, Doha, Qatar; 3 Department of Infectious Disease Epidemiology, Faculty of Epidemiology and Population Health, London School of Hygiene and Tropical Medicine, London, UK; 4 MRC Tropical Epidemiology Group, London School of Hygiene and Tropical Medicine GB, London, UK; 5 Department of Population Health Sciences, Weill Cornell Medicine, Cornell University, New York, NY USA

**Keywords:** Infectious diseases, Predictive markers

## Abstract

This study investigated herpes simplex virus type 2 (HSV-2) seroprevalence utility as a predictor of HIV epidemic potential among female sex workers (FSWs) globally. We updated and analyzed a systematically-assembled database for paired HSV-2 and HIV seroprevalence measures among FSWs. The study identified 231 paired HSV-2/HIV prevalence measures from 40 countries. The pooled mean HIV prevalence using meta-analysis increased from 3.7% (95% CI 0.3–9.9%) among populations of FSWs with HSV-2 prevalence < 25% to 18.7% (95% CI 14.1–23.8%) among those with HSV-2 prevalence 75–100%. HIV prevalence was negligible in FSWs with HSV-2 prevalence ≤ 20% suggesting a threshold effect. Multivariable meta-regressions explained > 65% of HIV prevalence variation, and identified a strong positive HSV-2/HIV association. Compared to populations of FSWs with HSV-2 prevalence < 25%, adjusted odds ratios (AORs) of HIV infection increased from 2.8 (95% CI 1.2–6.3) in those with HSV-2 prevalence 25–49%, to 13.4 (95% CI 6.1–29.9) in those with HSV-2 prevalence 75–100%. HSV-2 is a strong predictor of HIV epidemic potential among FSWs. HSV-2 prevalence of 25–49% indicates potential for intermediate-intensity HIV epidemics, with higher levels indicative of large epidemics. HSV-2 surveillance could inform HIV preparedness in countries where HIV prevalence among FSWs is still limited or at zero-level.

## Introduction

Female sex workers (FSWs) continue to be a vulnerable and stigmatized population that is disproportionately affected by HIV^1–3^. Although FSWs generally constitute a small proportion of the total adult female population, typically less than 1%, this translates to millions of women globally that are at high risk of HIV infection and in need of prevention or treatment services^4,5^.

In resource-limited settings, HIV prevalence among FSWs is estimated at an average of 12%, with odds of infection being 14-fold higher than among women in the general population^2^. Despite their increased risk, access to testing and linkage to treatment is often suboptimal, and could be even lower than that of women in the general population^6^. Until recently, HIV prevalence among FSWs in the World Health Organization (WHO) Eastern Mediterranean Region (EMRO) has been persistently very low^2^, with the exception of Djibouti and South Sudan where the epidemic is established at ~ 20%^5,7^. Over the last decade, however, epidemics emerged in this population in a number of EMRO countries^5^. While HIV prevalence remains low, it has been growing rapidly, by as much as ~ 15% per year^5^, with the potential for further growth being unknown. Epidemic potential is also unknown for half of EMRO countries where studies have consistently assessed HIV prevalence among FSWs at zero or negligible levels, but where documented overlap with other at-risk populations may create opportunities for seeding epidemics^5^.

Predicting HIV epidemic potential, that is the level that HIV prevalence can reach in a population, is essential for informing program development and resource allocation^8^. One approach is to use self-reported sexual risk behavior data. The latter, however, is limited by reporting bias, recall bias, limitations in value of ego-centric data to map level of risk in the sexual network, poor representability due to insufficient integrated bio-behavioral surveillance surveys (IBBSS), and lack of standardization across studies^5,9–12^. Since herpes simplex virus type 2 (HSV-2) is almost exclusively sexually transmitted, is more transmissible than HIV, and produces long-lasting antibodies, it has been used as a biological marker of sexual risk and objective indicator of the risk of exposure to HIV^8,9,13–15^. It is also believed, based on observational evidence, that there is an epidemiologic synergy between HSV-2 and HIV infection^16–18^, though recent evidence has casted doubt about this synergy^19^. Earlier analyses using empirical data as well as mathematical modeling also supported the utility of HSV-2 in predicting HIV epidemic potential^8,9,20^.

Limited HIV prevalence is often observed among FSWs in various settings suggesting that the virus may not yet have been introduced in commercial heterosexual sex networks, or may not have had sustainable transmission upon introduction^2,5^. In situations where HIV prevalence has been repeatedly assessed at zero or negligible levels, such as for several EMRO countries, periodic IBBSS for HIV surveillance, though desirable, is often (incorrectly) perceived as unnecessary^21–23^. Testing for other sexually transmitted infections (STIs) such as HSV-2 are also typically not incorporated in HIV surveillance activities^21,22,24^. However, the recent emergence and steady growth of HIV epidemics among FSWs in different EMRO countries, after years of limited or no prevalence, advocate for the relevance and urgency of collecting such data to enable assessment of HIV epidemic potential in these settings^5^.

This study systematically reviews paired HSV-2 and HIV (antibody) prevalence data among FSWs, globally, and analyzes these data to investigate use and utility of HSV-2 as a predictor of HIV prevalence and epidemic potential among FSWs by (1) estimating the pooled mean HIV prevalence at various HSV-2 prevalence levels, and (2) determining the magnitude of the HSV-2/HIV ecological association in light of regional, temporal, and condom use differences among FSWs.

## Results

### Search results and scope

**Figure 1:**
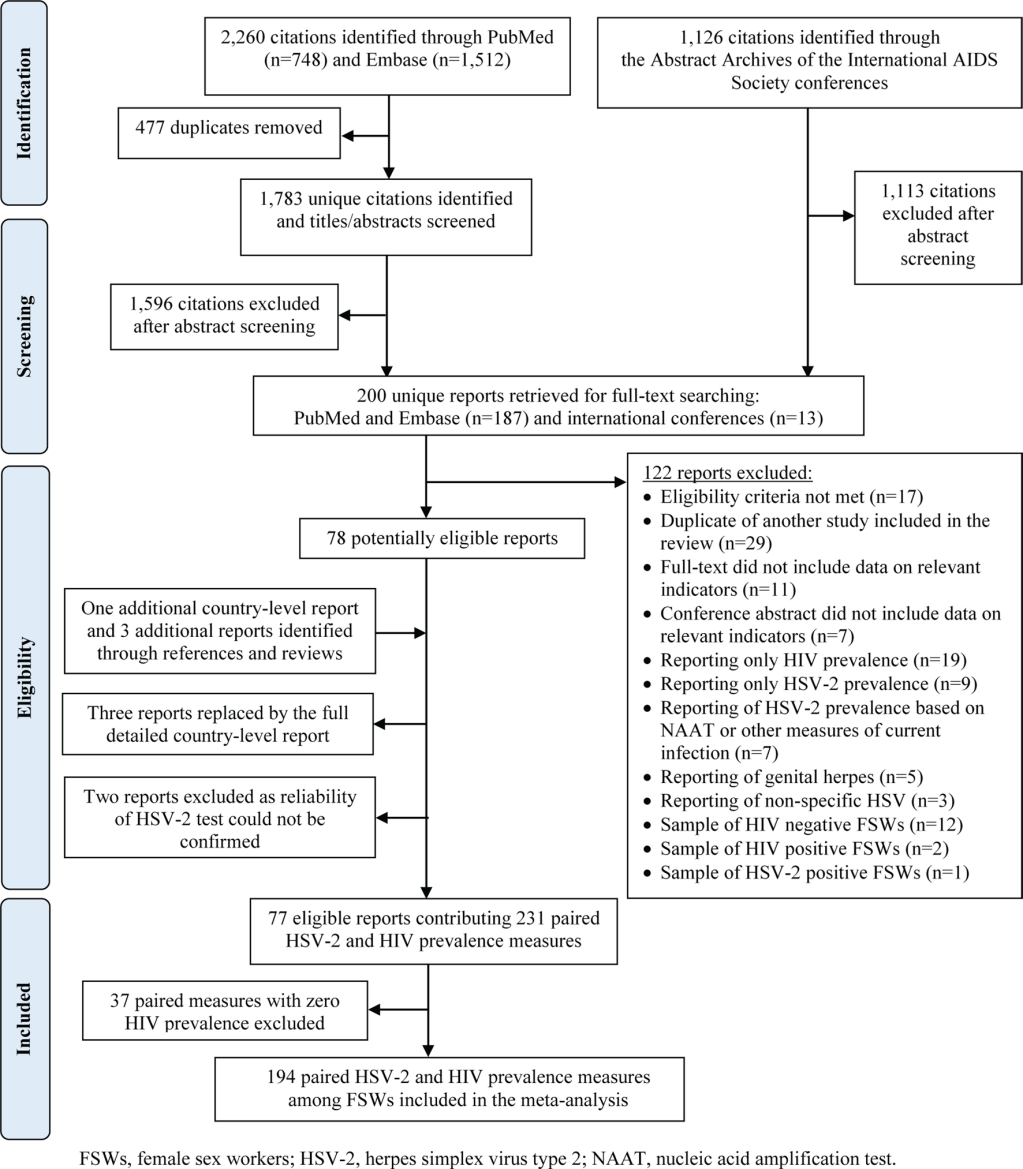
Flow chart presenting the process of study selection following PRISMA guidelines^47^.

The systematic search identified a total of 3386 citations, which after removing duplicates and screening, yielded 78 eligible reports (Fig. 1). Hand searching of the reference lists of eligible reports and reviews yielded three additional articles, and one comprehensive country-level public health report from India^25^ that replaced three other full-texts^26–28^. Two reports were further excluded after consulting with Professor Rhoda Ashley-Morrow, an expert advisor in HSV-2 diagnostics, because the reliability of HSV-2 serologic testing could not be confirmed^29,30^. In total, 77 reports comprising 231 paired HSV-2 and HIV prevalence measures among FSWs, from 40 countries, were eligible for inclusion. These contributed to the database generated through our earlier systematic review^20^ a total of 63 additional paired HSV-2 and HIV prevalence measures from 17 recent reports. Identified measures dated from 1988–2018 and are tabulated in Table S1 of Supplementary Information (SI) based on WHO regional classification [Region of the Americas (AMRO), African Region (AFRO), EMRO, European Region (EURO), South-East Asia Region (SEARO), and Western Pacific Region (WPRO)].

**Figure 2:**
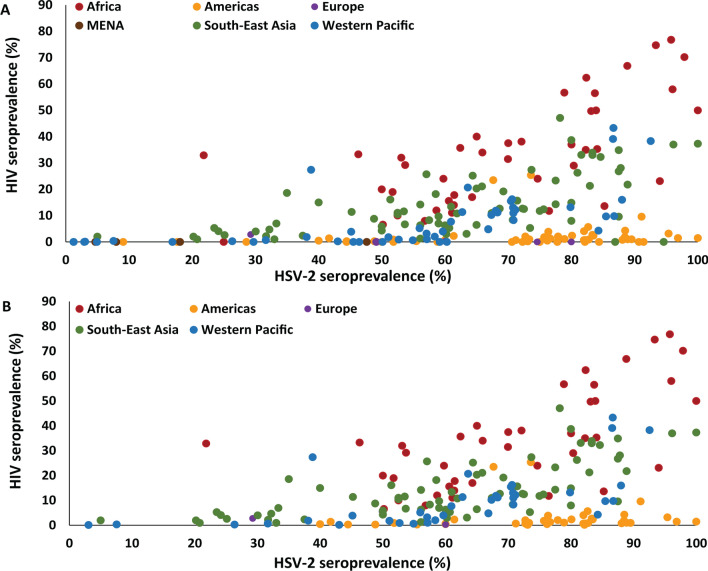
Scatterplot showing the global distribution of the paired herpes simplex type 2 (HSV-2) and HIV prevalence measures among female sex workers. (**A**) Distribution of *all* measures identified through the systematic review and (**B**) distribution of measures included in the analysis after excluding measures with zero HIV prevalence.

As the focus of this work is on examining the association between the two infections, it was pre-decided to restrict the analysis to settings where HIV has been introduced; we therefore excluded 37 paired measures with zero HIV prevalence from further analysis. After excluding measures with zero HIV prevalence, analysis was performed on a total of 194 paired measures from 33 countries (Fig. S1 of SI). India contributed the largest number of measures (n = 58; 29.9%), followed by China (n = 37, 19.1%), then Peru (n = 19; 9.8%). The distribution of measures across world regions is illustrated in Fig. 2A,B. The highest data contribution was for SEARO (n = 71; 36.6%), followed by AFRO and AMRO (each with n = 41; 21.1%), WPRO (n = 38; 19.6%), and lastly EURO (n = 3; 1.6%). There were only four studies from EMRO, all of which reported zero HIV prevalence, and thus were excluded from analysis.

### Overview of the distribution of HIV prevalence by HSV-2 prevalence

Table 1 summarizes HIV prevalence data among FSWs, stratified by HSV-2 prevalence. Globally, among FSWs with HSV-2 prevalence < 25%, the median HIV prevalence was 2.0% (n = 8; range = 0.1–32.7%), increased slightly to 2.5% (n = 23; range = 0.2–33.3%) with HSV-2 prevalence 25–49%, then increased sharply to 10.8% (n = 92; range = 0.2–39.7%) with HSV-2 prevalence 50–74%, and to 14.9% (n = 71; range = 0.2–76.8%) with HSV-2 prevalence 75–100%. The scatterplots illustrating the distribution of the paired HSV-2 and HIV prevalence measures further suggested a threshold effect with limited HIV prevalence at HSV-2 prevalence ≤ 20% (Fig. 2A; n = 13; median = 0.0; range = 0.0–2.0).

**Table 1:** Results of meta-analyses on studies reporting HIV prevalence among female sex workers stratified by HSV-2 prevalence levels.

HSV-2 prevalence^a^	Studies	Samples		HIV prevalence		Pooled mean HIV prevalence		Heterogeneity measures		
	N	Tested	HIV positive	Median (%)	Range (%)	(%)	95% CI	Q (*p* value)^c^	I^2^^d^ (%; 95% CI)	Prediction interval^e^ (95%)
**African region**										
< 25%	1^b^	220	72	32.7	–	–	–	–	–	–
25–49%	1^b^	54	18	33.3	**–**	–	–	**–**	**–**	**–**
50–74%	21	6895	1711	20.0	6.6–39.7	22.2	17.6–27.1	418.7 (*p* < 0.01)	95.2 (93.8–96.3)	4.5–47.6
75–100%	18	5829	2670	50.0	11.8–76.8	47.7	39.4–55.9	614.1 (*p* < 0.01)	97.2 (96.5–97.8)	14.1–82.5
Total	41	12,998	4471	32.7	6.6–76.8	33.1	27.8–38.7	1696.8 (*p* < 0.01)	97.6 (97.3–98.0)	5.2–70.0
**Other WHO regions**										
< 25%	7	2190	35	2.0	0.1–5.3	1.7	0.3–3.8	47.9 (*p* < 0.01)	87.5 (76.5–93.3)	0.0–12.0
25–49%	22	8280	580	2.5	0.2–27.4	3.9	1.6–7.1	877.1 (*p* < 0.01)	97.6 (97.1–98.1)	0.0–27.8
50–74%	71	28,935	2521	7.7	0.2–27.4	7.5	5.9–9.2	1954.2 (*p* < 0.01)	96.4 (95.9–96.9)	0.0–26.3
75–100%	53	15,243	2222	9.5	0.2–47.1	11.3	8.0–15.2	2607.0 (*p* < 0.01)	98.0 (97.7–98.2)	0.0–48.1
Total	153	54,648	5358	5.9	0.1–47.1	7.8	6.4–9.3	6130.8 (*p* < 0. 01)	97.5 (97.3–97.7)	0.0–33.2
**Global**										
< 25%	8	2410	107	2.0	0.1–32.7	3.7	0.3–9.9	247.7 (*p* < 0.01)	97.2 (95.9–98.1)	0.0–37.2
25–49%	23	8334	598	2.5	0.2–33.3	4.5	2.0–7.9	911.0 (*p* < 0.01)	97.6 (97.0–98.0)	0.0–29.6
50–74%	92	35,830	4232	10.8	0.2–39.7	10.1	8.2–12.3	3633.0 (*p* < 0.01)	97.5 (97.2–97.7)	0.0–36.0
75–100%	71	21,072	4892	14.9	0.2–76.8	18.7	14.1–23.8	5844.9 (*p* < 0.01)	98.8 (98.7–98.9)	0.0–69.2
Total	194	67,646	9829	10.1	0.1–76.8	11.8	9.9–13.9	12,598.5 (*p* < 0.01)	98.5 (98.4–98.6)	0.0–49.3

CI, confidence interval; HSV-2, herpes simplex virus type 2.

^a^Excluding 37 studies with zero HIV prevalence.

^b^Meta-analysis not possible for a single study.

^c^Q: the Cochran’s Q statistic is a measure assessing the existence of heterogeneity in effect size (here, HIV prevalence) across studies.

^d^I^2^: a measure assessing the magnitude of between-study variation that is due to differences in effect size (here, HIV prevalence) across studies rather than chance.

^e^Prediction interval: a measure estimating the 95% interval of the distribution of true effect sizes (here, HIV prevalence).

In AFRO, HSV-2 prevalence among FSWs was > 50% in almost all studies. The median HIV prevalence was 20.0% (n = 21; range = 6.6–39.7%) with HSV-2 prevalence 50–74%, and 50.0% (n = 18; range = 11.8–76.8%) with HSV-2 prevalence 75–100%. In the other regions, the median HIV prevalence was 2.0% (n = 7; range = 0.1–5.3%) with HSV-2 prevalence < 25%, 2.5% (n = 22; range = 0.2–27.4%) with HSV-2 prevalence 25–49%, 7.7% (n = 71; range = 0.2–27.4%) with HSV-2 prevalence 50–74%, and 9.5% (n = 53; range = 0.2–47.1%) with HSV-2 prevalence 75–100%.

The median proportion of FSWs who inject drugs was 3.3% (n = 33; range = 0.0–81.9; Table S1 of SI). It was 1.2% (n = 11; range = 0.5–51.6%) in AMRO, 3.5% (n = 10; range = 0.0–3.9%) in SEARO, and 7.4% (n = 9; range = 1.3–81.9%) in WPRO. Meanwhile, there were no studies from AFRO, only one study from EURO reporting this proportion at 0%, and two studies from EMRO each reporting this proportion at 3.0%.

The median HSV-2 prevalence across these studies was assessed at 33.3% (range = 4.7–95.7) while the median HIV prevalence was assessed at 3.2% (range = 0.0–39.1%). In studies where the proportion of FSWs who inject drugs was < 5%, the median HSV-2 prevalence was 30.0% (range = 4.7–95.7%) while the median HIV prevalence was 2.0% (n = 19; 95% CI 0.0–9.6%). In studies where the proportion of FSWs who inject drugs was ≥ 5% but < 10%, the median HSV-2 prevalence was 70.8% (n = 7; range = 29.7–86.6%) while the median HIV prevalence was 5.2% (95% CI 0.0–39.1%). In studies where the proportion of FSWs who inject drugs was ≥ 10%, the median HSV-2 prevalence was 82.0% (n = 3; range = 72.9–92.6%) while the median HIV prevalence was 4.1% (95% CI 0.3–38.3%).

### Pooled mean HIV prevalence stratified by HSV-2 prevalence

The results of meta-analyses estimating the pooled mean HIV prevalence stratified by HSV-2 prevalence are presented in Table 1. Forest plots are shown in Fig. S2 of SI.

Across world regions, the pooled mean HIV prevalence was estimated at 3.7% (95% confidence interval (CI) = 0.3–9.9%) with HSV-2 prevalence < 25%, 4.5% (95% CI 2.0–7.9%) with HSV-2 prevalence 25–49%, 10.1% (95% CI 8.2–12.3%) with HSV-2 prevalence 50–74%, and 18.7% (95% CI 14.1–23.8%) with HSV-2 prevalence 75–100%.

Estimates in AFRO were higher at 22.2% (95% CI 17.6–27.1%) with HSV-2 prevalence 50–74%, and 47.7% (95% CI 39.4–55.9%) with HSV-2 prevalence 75–100%. In the rest of world regions, the pooled mean HIV prevalence was 1.7% (95% CI 0.3–3.8%) with HSV-2 prevalence < 25%, 3.9% (95% CI 1.6–7.1%) with HSV-2 prevalence 25–49%, 7.5% (95% CI 5.9–9.2%) with HSV-2 prevalence 50–74%, and 11.3% (95% CI 8.0–15.2%) with HSV-2 prevalence 75–100%.

There was evidence for heterogeneity in HIV prevalence in all meta-analyses: Cochran’s Q statistic *p* values were < 0.01, I^2^ was mostly > 90% indicating that most variability is due to true differences in HIV prevalence rather than chance, and prediction intervals were generally wide affirming heterogeneity.

### Association of HSV-2 with HIV prevalence

Table 2 shows the results of meta-regression analyses examining the association between HIV prevalence and HSV-2 prevalence among FSWs globally. In the univariable analyses, HSV-2 prevalence, WHO region, year of data collection, and proportion of FSWs reporting consistent condom use were associated with HIV prevalence at *p* value ≤ 0.2, and hence were included in the multivariable analysis.

**Table 2:** Results of meta-regression analyses assessing the association between HIV prevalence and HSV-2 prevalence among female sex workers globally.

Factors	Studies	Samples	Univariable analyses				Multivariable analysis-model 1			Multivariable analysis-model 2		
	Total n	Total n	OR (95% CI)	*p* value	F *p* value^a^	Adj. R^2^ (%)	AOR (95% CI)	*p* value	F *p* value^b^	AOR^c^ (95% CI)	*p* value	F *p* value^b^
**HSV-2 prevalence**												
< 25%	8	2410	1.0		< 0.01	10.8	1.0		< 0.01	–	–	–
25–49%	23	8334	1.4 (0.4–5.0)	0.60			2.8 (1.2–6.3)	0.01		–	–	–
50– 74%	92	35,830	4.0 (1.3–12.6)	0.02			5.2 (2.4–11.3)	< 0.01		–	–	–
75–100%	71	21,072	7.2 (2.3–22.7)	< 0.01			13.4 (6.1–29.9)	< 0.01		–	–	–
**HSV-2 prevalence**^c^												
	191	66,239	1.03 (1.02–1.04)	< 0.01	< 0.01	10.8	–	–	–	1.04 (1.03–1.05)	< 0.01	< 0.01
WHO region												
AMRO	41	12,037	1.0		< 0.01	48.5	1.0		< 0.01	1.0		< 0.01
AFRO	41	12,998	31.7 (19.0–53.0)	< 0.01			37.1 (23.2–59.4)	< 0.01		36.2 (23.6–55.7)	< 0.01	
EURO	3	718	1.3 (0.3–5.2)	0.70			3.5 (1.0–12.1)	0.05		5.5 (1.7–17.5)	< 0.01	
SEARO	71	24,047	8.5 (5.4–13.4)	< 0.01			11.2 (7.0–17.8)	< 0.01		12.5 (8.1–19.1)	< 0.01	
WPRO	38	17,846	3.8 (2.3–6.4)	< 0.01			5.8 (3.4–9.9)	< 0.01		6.2 (3.8–10.0)	< 0.01	
**Publication year**												
< 2000	15	5049	1.0		0.83	0.0	–	–	–	–	–	–
2000–2004	7	2368	1.2 (0.3–5.5)	0.80			–	–	–	–	–	–
2005–2009	56	13,855	1.7 (0.6–4.3)	0.30			–	–	–	–	–	–
2010–2014	99	40,760	1.4 (0.6–3.6)	0.44			–	–	–	–	–	–
2015– 2019	17	5614	1.2 (0.4–3.7)	0.81			–	–	–	–	–	–
Data collection year^d^												
< 1995	18	6478	1.0		0.11	1.8	1.0		0.15	1.0		0.17
1995–1999	14	2462	1.0 (0.3–3.1)	0.97			0.7 (0.3–1.5)	0.31		0.7 (0.3–1.3)	0.26	
2000–2004	61	15,736	1.0 (0.4–2.5)	0.93			1.0 (0.6–1.7)	0.98		1.0 (0.6–1.6)	0.85	
2005–2009	88	37,770	2.0 (0.9–4.6)	0.10			1.4 (0.8–2.5)	0.30		1.3 (0.8–2.3)	0.34	
2010–2014	13	5200	1.4 (0.4–4.6)	0.56			0.6 (0.3–1.3)	0.19		0.6 (0.3–1.3)	0.22	
**Sample size**												
< 200	52	5507	1.0		0.76	0.0	–	–	–	–	–	–
≥ 200	142	62,139	0.92 (0.54–1.56)	0.76			–	–	–	–	–	–
**Proportion of FSWs reporting consistent condom use**												
75–100%	77	31,462	1.0		0.09	2.1	1.0		0.09	1.0		0.08
50–74%	19	8129	0.4 (0.2–1.0)	0.04			1.1 (0.6–1.9)	0.79		1.2 (0.7–2.0)	0.55	
25–49%	31	6367	1.4 (0.7–2.7)	0.38			1.8 (1.0–3.2)	0.05		1.9 (1.1–3.2)	0.02	
< 25%	9	3715	1.1 (0.3–3.3)	0.91			0.7 (0.3–1.5)	0.31		0.8 (0.4–1.6)	0.51	
Unclear	58	17,973	0.7 (0.4–1.2)	0.19			1.3 (0.8–2.1)	0.30		1.2 (0.8–1.8)	0.47	

Adj, Adjusted; AFRO, African Region; AMRO, Region of the Americas; AOR, adjusted odds ratio; CI, confidence interval; EURO, European Region; FSWs, female sex workers; HSV-2, herpes simplex virus type 2; OR, odds ratio; SEARO, South-East Asia Region; WHO, World Health Organization; WPRO, Western Pacific Region.

Adjusted R^2^ is 65.3% in the multivariable model 1, and 70.6% in the multivariable model 2.

^a^Factors with *p* value ≤ 0.2 were eligible for inclusion in the multivariable analysis.

^b^Factors with *p* value ≤ 0.05 and those with 0.05 < *p* value ≤ 0.1 in the multivariable model were considered as showing, respectively, “strong” and “some” evidence for an association with HIV prevalence.

^c^Analysis of the association with HSV-2 prevalence as a linear term excluded three measures with HSV-2 prevalence ≤ 20% in light of the observed threshold effect.

^d^Missing values for year of data collection were imputed using data for year of publication adjusted by the median difference between year of publication and median year of data collection for studies with complete information.

The multivariable models, whether considering HSV-2 prevalence as a categorical variable (Model 1) or as a linear variable (Model 2), both showed strong evidence for an association with HSV-2 and WHO region (*p* value ≤ 0.05). Some evidence for an association, that is a *p* value between 0.05 and 0.1, was found for consistent condom use, but no evidence (*p* value > 0.1) was found for year of data collection. Models 1 and 2 explained, respectively, 65.3% and 70.6% of the variation in HIV prevalence.

Model 1 showed that, relative to FSWs with HSV-2 prevalence < 25%, odds of HIV infection were three-fold higher (95% CI 1.2–6.3) among those with HSV-2 prevalence 25–49%, five-fold higher (95% CI 2.4–11.3) among those with HSV-2 prevalence 50–74%, and 13-fold higher (95% CI 6.1–29.9) among those with HSV-2 prevalence 75–100%. Regional differences were identified, where compared to AMRO, odds were four-fold higher for EURO (95% CI 1.0–12.1), six-fold higher for WPRO (95% CI 3.4–9.9), 11-fold higher for SEARO (95% CI 7.0–17.8), and thirty-seven-fold higher for AFRO (95% CI 23.2–59.4). FSWs reporting 25–49% consistent condom use had twice higher odds of HIV infection compared to those reporting 75–100% consistent condom use (95% CI 1.0–3.2).

Similar results were found using Model 2. Here, however, a 1% increase in HSV-2 prevalence among FSWs, beyond the 20% threshold, was associated with a 4% increase in the odds of HIV infection (adjusted odds ratio (AOR) = 1.04, 95% CI 1.03–1.05).

Table 3 shows the results of meta-regression analyses excluding AFRO. Results were consistent with those for all regions (Table 2). In Model 1, relative to FSWs with HSV-2 prevalence < 25%, AORs were 4.0 (95% CI 1.7–9.8), 7.8 (95% CI 3.3–18.2), 19.1 (95% CI 7.9–46.1) among those with HSV-2 prevalence 25–49%, 50–74%, and 75–100%, respectively. The AOR in the linear association (Model 2) was 1.04 (95% CI 1.03–1.05).

**Table 3:** Results of meta-regression analyses assessing the association between HIV prevalence and HSV-2 prevalence among female sex workers globally but excluding the African Region.

Factors	Studies	Samples	Univariable analyses				Multivariable analysis-model 1			Multivariable analysis-model 2		
	Total n	Total n	OR (95% CI)	*p* value	F *p* value^a^	Adj. R^2^ (%)	AOR (95% CI)	*p* value	F *p* value^b^	AOR^c^ (95% CI)	*p* value	F *p* value^b^
**HSV-2 prevalence**												
< 25%	7	2190	1.0		< 0.01	8.6	1.0		< 0.01	–	–	–
25–49%	22	8280	1.9 (0.6–6.7)	0.29			4.0 (1.7–9.8)	< 0.01		–	–	–
50–74%	71	28,935	4.5 (1.5–14.0)	< 0.01			7.8 (3.3–18.2)	< 0.01		–	–	–
75–100%	53	15,243	6.2 (2.0–19.4)	< 0.01			19.1 (7.9–46.1)	< 0.01		–	–	–
**HSV-2 prevalence**^c^												
	150	53,241	1.02 (1.01–1.04)	< 0.01	< 0.01	7.1	–	–	–	1.04 (1.03–1.05)	< 0.01	< 0.01
WHO region												
AMRO	41	12,037	1.0		< 0.01	34.0	1.0		< 0.01	1.0		< 0.01
EURO	3	718	1.3 (0.3–5.6)	0.71			4.1 (1.2–14.6)	0.03		6.5 (2.0–21.2)	< 0.01	
SEARO	71	24,047	8.5 (5.3–13.6)	< 0.01			10.3 (6.3–16.9)	< 0.01		11.3 (7.1–17.8)	< 0.01	
WPRO	38	17,846	3.8 (2.2–6.6)	< 0.01			5.3 (3.0–9.5)	< 0.01		5.5 (3.2–9.4)	< 0.01	
**Publication year**												
< 2000	10	2920	1.0		0.02	5.4	–	–	–	–	–	–
2000–2004	4	734	0.7 (0.1–3.6)	0.63			–	–	–	–	–	–
2005–2009	36	10,101	1.3 (0.4–3.5)	0.67			–	–	–	–	–	–
2010–2014	93	37,170	2.4 (0.9–6.2)	0.08			–	–	–	–	–	–
2015–2019	10	3723	0.7 (0.2–2.7)	0.63			–	–	–	–	–	–
**Data collection year**^d^												
< 1995	12	3384	1.0		< 0.01	23.4	1.0		< 0.01	1.0		< 0.01
1995–1999	12	2059	1.2 (0.4–3.4)	0.76			0.5 (0.2–1.3)	0.15		0.5 (0.2–1.2)	0.12	
2000–2004	42	11,247	0.7 (0.3–1.7)	0.44			0.8 (0.4–1.6)	0.59		0.8 (0.4–1.5)	0.42	
2005–2009	81	34,649	3.6 (1.6–8.0)	< 0.01			1.7 (0.8–3.4)	0.17		1.6 (0.8–3.0)	0.18	
2010–2014	6	3309	0.6 (0.2–2.1)	0.39			0.3 (0.1–1.0)	0.04		0.4 (0.2–1.0)	0.06	
**Sample size**												
< 200	36	4422	1.0		0.38	0.0	–	–	–	–	–	–
≥ 200	117	50,226	1.3 (0.7–2.3)	0.38			–	–	–	–	–	–
**Proportion of FSWs reporting consistent condom use**												
75–100%	73	30,137	1.0		< 0.01	11.6	1.0		0.07	1.0		0.04
50–74%	16	5537	0.3 (0.1–0.6)	< 0.01			1.6 (0.8–3.1)	0.17		1.6 (0.9–2.9)	0.14	
25–49%	19	3967	0.9 (0.4–1.8)	0.69			2.7 (1.4–5.3)	< 0.01		2.7 (1.5–5.1)	< 0.01	
< 25%	3	988	0.3 (0.1–1.5)	0.15			1.1 (0.3–3.8)	0.88		1.3 (0.4–4.2)	0.62	
Unclear	42	14,019	0.3 (0.2–0.5)	< 0.01			1.6 (0.9–2.7)	0.11		1.4 (0.9–2.4)	0.16	

Adj, Adjusted; AMRO, Region of the Americas; AOR, adjusted odds ratio; CI, confidence interval; EURO, European Region; FSWs, female sex workers; HSV-2, herpes simplex virus type 2; OR, odds ratio; SEARO, South-East Asia Region; WHO, World Health Organization; WPRO, Western Pacific Region.

Adjusted R^2^ is 58.2% in the multivariable model 1, and 64.1% in the multivariable model 2.

^a^Factors with *p* value ≤ 0.2 were eligible for inclusion in the multivariable analysis.

^b^Factors with *p* value ≤ 0.05 and those with 0.05 < *p* value ≤ 0.1 in the multivariable model were considered as showing, respectively, “strong” and “some” evidence for an association with HIV prevalence.

^c^Analysis of the association with HSV-2 prevalence as a linear term excluded three measures with HSV-2 prevalence ≤ 20% in light of the observed threshold effect.

^d^Missing values for year of data collection were imputed using data for year of publication adjusted by the median difference between year of publication and median year of data collection for studies with complete information.

## Discussion

Motivated by the concept of using current HSV-2 prevalence in a population as a proxy biomarker of future HIV prevalence in that population^8,9,20^ and its relevance to HIV preparedness, this study assessed the utility of HSV-2 as a predictor of HIV epidemic potential among FSWs through a global systematic analysis of empirical paired HSV-2 and HIV prevalence measures. We found strong evidence for an association between HIV and HSV-2 prevalence, even after accounting for potential confounders such as region, temporal trend, and condom use (Tables 2 and 3). HIV prevalence was negligible at HSV-2 prevalence ≤ 20% (Fig. 2), but increased steadily with higher HSV-2 prevalence suggesting a threshold effect—the odds of HIV infection doubled with a 25% increase in HSV-2 prevalence (Tables 1 and 2). These findings demonstrate that in populations where HIV prevalence is still limited, but has potential to grow, HSV-2 prevalence can be used to provide a prediction of future HIV prevalence.

The hierarchy of HIV prevalence among FSWs was evident even in the context of Africa’s general population HIV epidemics (Table 1). Outside the African Region, HSV-2 prevalence among FSWs of 25–49% was indicative of the potential for intermediate-intensity HIV epidemics with an HIV prevalence of ~ 5% or less. For FSW populations with HSV-2 prevalence ≥ 50%, HIV prevalence was higher and often exceeded 10%. Our findings based on analysis of empirical data substantiate mathematical modeling analyses predicting quantitatively such an association^8,9^, which also appears to exist for other populations^20^. The modeling analyses simulating HSV-2 and HIV propagation along diverse sexual networks demonstrated that HSV-2 prevalence ≥ 50% is indicative of substantial sexual risk behavior, sufficient to sustain large HIV epidemics in a sexual network^9^. In contrast, HSV-2 prevalence < 20% in a sexual network is indicative of low sexual risk behavior that is not likely to sustain an epidemic (a “threshold effect”)^8^. Both of these modeling predictions were confirmed in the present study through analysis of actual empirical data (Table 1).

After decades of virtually zero HIV prevalence^2^, EMRO has recently seen emergence of HIV epidemics among FSWs in several countries^5^. However, and despite an apparently rapid epidemic growth, HIV prevalence in FSWs remains overall at low levels^5^. It is unfortunate that there were too few HSV-2 prevalence measures among FSWs in this region to predict HIV epidemic potential (Table S1 of SI)^24^. Available measures indicated also relatively low HSV-2 prevalence, often below 20% (Table S1 of SI)^24^, the apparent threshold for a significant HIV epidemic (Fig. 2). HSV-2 prevalence in the general population in EMRO also appears to be low, and overall lower than that in other regions^8,31^. Indeed, a recent global assessment^32^ estimated HSV-2 prevalence among women in the general population at 7.6% in EMRO, 9.6% in SEARO, 10.7% in EURO, 14.6% in WPRO, 24.0% in AMRO, and 43.9% in AFRO, whereas median HSV-2 prevalence among FSWs in our study was > 50% in all regions aside from EMRO. This suggests that HIV prevalence may not grow to reach considerable levels in many FSW populations in EMRO, and possibly will persist at levels close to zero HIV prevalence. Having said so, this region could largely benefit from integrating testing for HSV-2 in HIV surveillance activities. However, much more data on HSV-2 prevalence are needed before we can assess HIV epidemic potential among FSWs in this region with meaningful confidence.

Several other findings emerged from this study. There was regional variation in HIV prevalence that could not be captured by HSV-2 prevalence, especially so for the African Region (Table 2), but also outside Africa (Table 3). This finding suggests that other factors may differentially impact each of HSV-2 and HIV prevalence, and that these should be accounted for to better describe the HIV/HSV-2 association. This is also supported by modeling analyses that demonstrated that, while some sexual network statistics affect HSV-2 and HIV transmission similarly, others can affect them differentially^9^. A plausible explanation relates to HIV having lower infectiousness and shorter acute infection duration, therefore facing more difficulty in propagating within sexual networks compared to HSV-2^9^. For instance, while concurrency (mean number of current sexual partners) is a strong predictor of both HSV-2 and HIV prevalence, clustering within a sexual network (or high exposure within specific circles), provides a higher chance for HIV to spread, but limits HSV-2 from reaching farther nodes in the wider sexual network^9^. Meanwhile, higher degree correlation, that is broad connectivity between sexual partnerships, appears to favor HSV-2 spread, but not HIV^9^. This suggests that, despite the strength of the association, HSV-2 cannot be used as the sole predictor of HIV epidemic potential.

Our findings indicated only a small role for self-reported condom use in predicting HIV prevalence (Tables 2 and 3), suggesting that such self-reported behavioral measures may not carry meaningful explanatory power, and affirming documented issues in self-reported measures^11,12,33^.

Our study has limitations. There was variability in the number of paired HSV-2/HIV prevalence measures among FSWs across regions, thus limiting our ability to perform further stratified, region-specific, analyses. For instance, there was an insufficient number of studies from EURO to warrant meaningful analysis and interpretation, and no studies from EMRO. Our regional estimates may have also been biased by some countries having larger data contributions (that is more or larger sample size studies) than others, but meta-regression analyses did not identify an association with study sample size. There was also heterogeneity in HIV prevalence, as commonly seen in observational studies assessing prevalence^5,34^. The latter, however, was (mostly) explained through the meta-regression analyses, which affirmed HSV-2 prevalence as an independent contributor to this heterogeneity (Tables 2 and 3). Only a handful of studies reported age-related data, and these varied immensely in the type of reported measure, thus constraining age inclusion in the analysis.

A number of studies did not report data on condom use among FSWs, and very few reported coverage for other interventions to warrant their inclusion in the analyses. For example, only one study reported antiretroviral therapy (ART) coverage (Table S1 of SI), which presumably could affect the association between HIV and HSV-2 prevalence. This being said, most studies were conducted before the mass scale up of ART (Table 2), and thus ART is unlikely to have affected the observed association in the current analysis but may impact future analyses on future data examining this association. Few studies also reported data on current injecting drug use, a non-sexual mode of HIV transmission, with overall no major differences across regions. The latter however is unlikely to have affected the observed HSV-2/HIV association given that the median fraction of FSWs currently injecting drugs is < 5% (Table S1 of SI). Our findings also showed that even in studies where the proportion of FSWs who inject drugs was ≥ 5%, HSV-2 prevalence was substantial with a median of 72%, likely given the nature of the study population and/or the likelihood of exchanging sex for drugs.

The association between HIV prevalence and HSV-2 prevalence is likely non-linear, although the distribution of measures (Fig. 2) and an earlier mathematical modeling analysis^8^ suggested that this association may not be far from linearity (above the threshold effect). This implies that our AOR for the HIV/HSV-2 (linear term) association should be interpreted with caution as an estimate for the average increase in odds of HIV prevalence per 1% increase in HSV-2 prevalence beyond the 20% threshold. While HSV-2 prevalence was probably at endemic equilibrium given infection circulation in human populations for centuries, HIV prevalence may not have been at equilibrium, but we were unable to account for the HIV epidemic phase in the analysis^17^. Despite these limitations, the parsimonious multivariable meta-regression models explained > 65% of the variation in HIV prevalence supporting the inferences drawn in this study.

In conclusion, we demonstrated an association between HSV-2 prevalence and HIV prevalence among FSWs that can be utilized in assessing HIV epidemic potential in this at-risk population. We also demonstrated the relevance of integrating testing for HSV-2 in HIV surveillance activities targeting this population, especially in settings where HIV prevalence among them is still at negligible or low level. Our findings stress the need for HSV-2 testing in future surveillance efforts, notably in IBBSS surveys, as a tool to inform HIV preparedness and resource allocation, particularly in countries where HIV epidemic potential among key populations remains unknown. Such data is essential to avoid the costly implications of emerging HIV epidemics and to ensure that countries are still “on track” towards ending AIDS^35^.

## Methods

### Data sources and selection methods

We updated a database of paired HSV-2 and HIV prevalence measures, retrieved through an earlier systematic review^20^, by conducting a new search focused on FSWs, on September 3rd, 2019, using broad MeSH/Emtree and free text terms for “sex work”, “women”, “HSV-2”, and “HIV” (search criteria in Box S1 of SI). Paired measures eligible for inclusion were identified through a systematic review process following Cochrane Collaboration guidelines^36^. Briefly, PubMed, Embase, and the abstract archives of International AIDS Society conferences were surveyed. Citations were screened for duplication, and then for relevance using Endnote (Thomson Reuters, USA). Full-texts of articles deemed relevant or potentially relevant underwent further screening, and paired measures for HSV-2 and HIV antibody prevalence (seroprevalence), based on primary data, were identified and extracted along with key information on study population characteristics, year(s) of data collection, year of publication, country of origin/survey, number tested and number positive for HSV-2 and HIV infections, diagnostic tests used for infections’ ascertainment, proportion of FSWs who inject drugs, proportion of infected FSWs on ART, and proportion of FSWs reporting consistent condom use. The latter was assessed primarily using self-reported condom use at last sex with client, or alternatively using self-reported “consistent/regular” condom use or condom use “all the time” during commercials sex acts (extraction list in Box S2 of SI).

### Plan of analysis

#### Descriptive analysis

Scatterplots were generated to illustrate the distribution of paired HSV-2 and HIV prevalence measures among FSWs across world regions. Countries’ regional classification was based on the WHO regional definition (WHO classification in Box S3 of SI)^37^. Maps showing countries’ data contribution were generated using Tableau Desktop v.10.1^38^. Studies were classified into four categories based on HSV-2 prevalence level among FSWs (< 25%, 25–49%, 50–74%, and 75–100%). Descriptive statistics of the reported HIV prevalence measures were then calculated stratified by HSV-2 prevalence category.

#### Meta-analysis

Forest plots were used to visualise estimates of HIV prevalence and 95% CIs for each HSV-2 stratum. The pooled mean HIV prevalence and associated 95% CIs were estimated, for different HSV-2 strata, using random-effects meta-analysis. Here, variances of HIV prevalence measures were first stabilized using a Freeman-Tukey type arcsine square-root transformation^39,40^. Prevalence measures were then weighted using the inverse-variance method^40,41^, and subsequently pooled using a DerSimonian-Laird random-effects model^42^ to account for sampling variation and true between-study heterogeneity^43^.

Heterogeneity across HIV prevalence measures was assessed, with and without considering HSV-2 stratification, using: Cochran’s Q statistic to confirm existence of heterogeneity across prevalence measures, I^2^ to quantify magnitude of variation that is due to true differences in prevalence across studies rather than chance, and prediction interval to estimate the 95% interval of the distribution of true prevalence measures^43,44^. Additional meta-analyses contrasting the African Region to the rest of world regions were performed, for relevance, as almost all HSV-2 prevalence measures in this region were > 50% (in contrast to the other regions), and considering the unique HIV epidemic history in this part of the world^1^.

Meta-analyses were implemented in R v.3.4.2^45^.

#### Meta-regression

Random-effects meta-regression analyses were conducted to assess whether HSV-2 prevalence can be used as a predictor of HIV prevalence among FSWs. Covariates, considered a priori, included: WHO region (AMRO, AFRO, EMRO, EURO, SEARO, and WPRO), publication year (< 2000, 2000–2004, 2005–2009, 2010–2014, 2015–2019), data collection year (< 1995, 1995–1996, 2000–2004, 2005–2009, 2010–2014), study sample size (< 200, ≥ 200), and proportion of FSWs reporting consistent condom use (< 25%, 25–49%, 50–74%, 75–100%, unclear). Proportion of FSWs who inject drugs could not be factored in our analysis given the low number of studies and heterogeneity across measures (Table S1 of SI). The proportion of infected FSWs on ART also could not be factored in our analysis as only a single measure was identified (Table S1 of SI). Missing values for year of data collection were imputed using data for year of publication adjusted by the median difference between year of publication and year of data collection (for studies with complete information). Meta-regression analyses were performed using two scenarios including and excluding AFRO. Meta-regressions estimated the *odds ratios* of HIV infection assuming that the probability of HIV infection for a given population is equal to that of HIV prevalence in this population.

Factors associated with HIV prevalence at *p* value ≤ 0.20 in univariable analysis were eligible for inclusion in the multivariable analysis. Two multivariable models were considered using HSV-2 prevalence as a categorical variable, or as a linear term after excluding HSV-2 prevalence ≤ 20% given observed threshold effect. In the multivariable model, a *p* value of ≤ 0.05 for any factor indicated strong evidence for an association with HIV prevalence, while 0.05 < *p* value ≤ 0.1 indicated some evidence for an association with prevalence.

Meta-regressions were implemented in Stata/SE v.16^46^.

## Supplementary information

Supplementary Information.
