# Supplementary material for: HSV-2 as a biomarker of HIV epidemic potential in female sex workers: meta-analysis, global epidemiology and implications
Source: Sci Rep. 2020 Nov 9;10:19293. doi: 10.1038/s41598-020-76380-z (PMC7652938; doi:10.1038/s41598-020-76380-z)
Supplement: Supplementary file 1 — Supplementary Information. [file 41598_2020_76380_MOESM1_ESM.docx]

**Supplementary Information**

**HSV-2 as a biomarker of HIV epidemic potential in female sex workers: meta-analysis, global epidemiology and implications**

Hiam Chemaitelly^1,2,3*^, Helen A. Weiss^4^, Laith J. Abu-Raddad^1,2,5^

^1^ Infectious Disease Epidemiology Group, Weill Cornell Medicine-Qatar, Cornell University, Qatar Foundation – Education City, Doha, Qatar

^2^ World Health Organization Collaborating Centre for Disease Epidemiology Analytics on HIV/AIDS, Sexually Transmitted Infections, and Viral Hepatitis, Weill Cornell Medicine–Qatar, Cornell University, Qatar Foundation – Education City, Doha, Qatar

^3^ Department of Infectious Disease Epidemiology, Faculty of Epidemiology and Population Health, London School of Hygiene and Tropical Medicine GB, London, United Kingdom

^4^ MRC Tropical Epidemiology Group, London School of Hygiene and Tropical Medicine, London, United Kingdom

^5^ Department of Healthcare Policy & Research, Weill Cornell Medicine, Cornell University, New York, New York, USA

**^*^ Corresponding author:** Hiam Chemaitelly, Weill Cornell Medicine-Qatar, Qatar Foundation-Education City, P.O. Box 24144, Doha, Qatar. Telephone: +(974) 4492-8443. Fax: +(974) 4492-8422. E-mail: [hsc2001@qatar-med.cornell.edu](mailto:hsc2001@qatar-med.cornell.edu)

**Table S1.** Paired HSV-2 and HIV prevalence measures among female sex workers identified in the systematic review.

| **Country** | **Short citation** | **Data collect. year(s)** | **Population characteristics** | **Site** | **Tested HSV-2 (n)** | **HSV-2 prev (%)** | **Tested HIV (n)** | **HIV prev (%)** | **ART cov (%)** | **Consistent condom use**^a^ **(%)** | **Prop who inject drugs (%)** |
| --- | --- | --- | --- | --- | --- | --- | --- | --- | --- | --- | --- |
| **AFRO (n=42)** |  |  |  |  |  |  |  |  |  |  |  |
| Burkina Faso | Low, 2011^1^ | 2003-05 | Professional FSWs | Community | 689 | 62.4 | 765 | 35.7 | 9.5 | 64.0 | -- |
| Burkina Faso | Nagot, 2003^2^ | 1998-02 | FSWs in Bobo-Dioulasso | Community | 540 | 66.0 | 562 | 34.0 | -- | -- | -- |
| Burkina Faso | Nagot, 2007^3^ | 2002-03 | FSWs in Bobo-Dioulasso | STI clinic | 273 | 70.1 | 273 | 31.5 | -- | 100.0 | -- |
| Congo | Nzila, 1991^4^ | 1988 | Hotel/home/street-based FSWs in Kinshasa | Community | 265 | 82.3 | 1226 | 35.0 | -- | 8.0 | -- |
| Congo | Vandepitte, 2007^5^ | 2002 | Hotel-based FSWs | STI clinic | 17 | 76.5 | 17 | 11.8 | -- | -- | -- |
| Congo | Vandepitte, 2007^5^ | 2002 | Home-based FSWs | STI clinic | 146 | 74.7 | 146 | 24.0 | -- | -- | -- |
| Congo | Vandepitte, 2007^5^ | 2002 | Street-based FSWs | STI clinic | 10 | 50.0 | 10 | 20.0 | -- | -- | -- |
| Congo | Vandepitte, 2007^5^ | 2002 | Homeless FSWs | STI clinic | 40 | 52.5 | 40 | 10.0 | -- | -- | -- |
| Congo | Vandepitte, 2007^5^ | 2002 | Clandestine FSWs | STI clinic | 289 | 50.2 | 289 | 6.6 | -- | -- | -- |
| Eritrea | Ghebrekidan, 1999^6^ | 1995 | Registered FSWs in Massawa | Health center | 107 | 80.0 | 107 | 29.0 | -- | -- | -- |
| Ethiopia | Holt, 2003^7^ | 1992 | FSWs from Fandinka and Amon | Community | 203 | 65.0 | 209 | 40.0 | -- | -- | -- |
| Guinea | Aho, 2014^8^ | 2005-06 | FSWs in Conakry | Health center | 201 | 84.1 | 223 | 35.3 | -- | 98.7 | -- |
| Guinea | Diakite, 2006^9^ | -- | FSWs in Conakry | Unclear | 416 | 72.1 | 417 | 38.1 | -- | -- | -- |
| Kenya | Vandenhoudt, 2013^10^ | 1997 | FSWs recruited at workplace in Kisumu | Community | 286 | 93.4 | 296 | 74.7 | -- | 49.8 | -- |
| Kenya | Vandenhoudt, 2013^10^ | 2008 | FSWs recruited through RDS in Kisumu | Community | 479 | 83.8 | 479 | 56.5 | -- | 75.5 | -- |
| Mozambique | Lafort, 2008^11^ | -- | FSWs at a reproductive health clinic in Tete | Health center | 350 | 83.1 | 350 | 49.7 | -- | 92.5 | -- |
| Nigeria | Dada, 1998^12^ | 1990-91 | Low class FSWs (low fee) | Community | 84 | 64.3 | 84 | 17.0 | -- | 0.0^c^ | -- |
| Nigeria | Dada, 1998^12^ | 1990-91 | Middle class FSWs (medium fee) | Community | 624 | 58.7 | 624 | 12.0 | -- | 0.0^c^ | -- |
| Nigeria | Dada, 1998^12^ | 1990-91 | Upper class FSWs (hotels/clubs) | Community | 88 | 56.8 | 88 | 8.0 | -- | 0.0^c^ | -- |
| Nigeria | Eltom, 2002^13^ | 1991-94 | FSWs from brothels or hotels in Lagos | Brothel/hotel | 863 | 60.60 | 863 | 15.6 | -- | -- | -- |
| Rwanda | Braunstein, 2011^14^ | -- | FSWs in Kigali | Community | 800 | 59.80 | 800 | 24.0 | -- | 74.0 | -- |
| Senegal | Kane, 2009^15^ | 2006 | FSWs in Dakar aged <20 years | Unclear | 12 | 25.0 | 12 | 0.0^b^ | -- | -- | -- |
| Senegal | Kane, 2009^15^ | 2006 | FSWs in Dakar aged 20-24 years | Unclear | 54 | 61.1 | 54 | 11.1 | -- | -- | -- |
| Senegal | Kane, 2009^15^ | 2006 | FSWs in Dakar aged 25-29 years | Unclear | 88 | 85.2 | 88 | 13.6 | -- | -- | -- |
| Senegal | Kane, 2009^15^ | 2006 | FSWs in Dakar aged ≥30 years | Unclear | 450 | 94.0 | 450 | 23.1 | -- | -- | -- |
| South Africa | Malope, 2008^16^ | 2001 | FSWs in a mining town in Carletonville | Community | 95 | 95.8 | 95 | 76.8 | -- | -- | -- |
| South Africa | Ramjee, 2005^17^ | -- | FSWs near truck stops in Kwazulu Natal | Health center | 416 | 84.0 | 416 | 50.0 | -- | 11.2 | -- |
| Tanzania | Riedner, 2007^18^ | 2000 | FSWs in entertainment venues in Mbeya | Community | 753 | 88.8 | 753 | 66.9 | -- | -- | -- |
| Tanzania | Vu, 2018^19^ | 2013 | FSWs in Dar es Salaam, Tanzania | Community | 324 | 53.1 | 324 | 32.0 | -- | 30.0^c^ | -- |
| Tanzania | Vu, 2018^19^ | 2013 | FSWs in Iringa, Tanzania | Community | 220 | 21.8 | 220 | 32.9 | -- | 30.0^c^ | -- |
| Tanzania | Vu, 2018^19^ | 2013 | FSWs in Mbeya, Tanzania | Community | 244 | 53.7 | 244 | 29.2 | -- | 30.0^c^ | -- |
| Tanzania | Vu, 2018^19^ | 2013 | FSWs in Mwanza, Tanzania | Community | 350 | 51.7 | 350 | 19.0 | -- | 30.0^c^ | -- |
| Tanzania | Vu, 2018^19^ | 2013 | FSWs in Shinyanga, Tanzania | Community | 320 | 70.0 | 320 | 37.5 | -- | 30.0^c^ | -- |
| Tanzania | Vu, 2018^19^ | 2013 | FSWs in Tabora, Tanzania | Community | 228 | 61.4 | 228 | 14.0 | -- | 30.0^c^ | -- |
| Tanzania | Vu, 2018^19^ | 2013 | FSWs in Mara, Tanzania | Community | 205 | 61.5 | 205 | 17.8 | -- | 30.0^c^ | -- |
| Uganda | Vandepitte, 2011^20^ | 2009 | FSWs from red-light district in Kampala | Red-light district | 1026 | 80.0 | 1027 | 37.0 | -- | 60.0 | -- |
| Zimbabwe | Cowan, 2005^21^ | -- | FSWs aged ≤20 years near mines & farms | Community | 54 | 46.3 | 54 | 33.3 | -- | -- | -- |
| Zimbabwe | Cowan, 2005^21^ | -- | FSWs aged 21-25 years near mines & farms | Community | 90 | 78.9 | 90 | 56.7 | -- | -- | -- |
| Zimbabwe | Cowan, 2005^21^ | -- | FSWs aged 26-30 years near mines & farms | Community | 85 | 82.4 | 85 | 62.4 | -- | -- | -- |
| Zimbabwe | Cowan, 2005^21^ | -- | FSWs aged 31-35 years near mines & farms | Community | 47 | 97.9 | 47 | 70.2 | -- | -- | -- |
| Zimbabwe | Cowan, 2005^21^ | -- | FSWs aged 36-40 years near mines & farms | Community | 50 | 96.0 | 50 | 58.0 | -- | -- | -- |
| Zimbabwe | Cowan, 2005^21^ | -- | FSWs aged 41-45 years near mines & farms | Community | 30 | 100.0 | 30 | 50.0 | -- | -- | -- |
| **AMRO (n=57)** | |  |  |  |  |  |  |  |  |  |  |
| Belize | Alvarez Rodriguez, 2013^22^ | -- | FSWs in Belize | Community | 220 | 51.8 | 220 | 0.9 | -- | 81.3 | -- |
| Domin. Rep. | Koenig, 2007^23^ | 2004-05 | FSWs in Santo Domingo | Community | 482 | 76.3 | 482 | 3.9 | -- | 14.0 | -- |
| El Salvador | Creswell, 2010^24^ | 2008 | FSWs in San Salvadore | Community | 663 | 82.6 | 663 | 5.7 | -- | 74.5 | -- |
| El Salvador | Soto, 2007^25^ | 2001-02 | Brothel & mobile FSWs | Community | 130 | 95.7 | 484 | 3.2 | -- | 72.9 | 0.5 |
| Guatemala | Soto, 2007^25^ | 2001-02 | Brothel & mobile FSWs | Community | 522 | 88.6 | 511 | 4.3 | -- | 82.5 | 1.3 |
| Honduras | Morales-Miranda^26^ | 2006 | FSWs in 4 cities | Community | 808 | 61.4 | 811 | 2.3 | -- | 80.0 | -- |
| Honduras | Soto, 2007^25^ | 2001-02 | Brothel & mobile FSWs | Community | 416 | 91.1 | 493 | 9.6 | -- | 93.8 | 3.3 |
| Mexico | Uribe-Salas, 1999^27^ | 1993 | FSWs working in massage parlors | Community | 72 | 44.4 | 76 | 0.0^b^ | -- | 80.6^c^ | -- |
| Mexico | Uribe-Salas, 1999^27^ | 1993 | FSWs working in bars | Community | 339 | 55.5 | 364 | 0.3 | -- | 80.6^c^ | -- |
| Mexico | Uribe-Salas, 1999^27^ | 1993 | Street-based FSWs | Community | 346 | 78.9 | 362 | 1.1 | -- | 80.6^c^ | -- |
| Mexico | Uribe-Salas, 2003^28^ | 1998 | FSWs working in bars from Guatemala | Community | 191 | 89.5 | 195 | 1.0 | -- | -- | 0.8^de^ |
| Mexico | Uribe-Salas, 2003^28^ | 1998 | FSWs working in bars from El Salvador | Community | 75 | 90.7 | 76 | 0.0^b^ | -- | -- | 0.8^de^ |
| Mexico | Uribe-Salas, 2003^28^ | 1998 | FSWs working in bars from Honduras | Community | 85 | 70.6 | 86 | 0.0^b^ | -- | -- | 0.8^de^ |
| Mexico | Uribe-Salas, 2003^28^ | 1998 | FSWs working in bars from Mexico | Community | 109 | 88.1 | 121 | 0.8 | -- | -- | 0.8^de^ |
| Nicaragua | Delgado, 2011^29^ | 2001-09 | FSWs in Managua | Community | 613 | 75.7 | 613 | 1.8 | -- | 89.9^c^ | -- |
| Nicaragua | Delgado, 2011^29^ | 2001-09 | FSWs in Chinandega | Community | 212 | 83.5 | 211 | 2.4 | -- | 89.9^c^ | -- |
| Nicaragua | Soto, 2007^25^ | 2001-02 | Brothel & mobile FSWs | Community | 454 | 82.1 | 460 | 0.2 | -- | 56.6 | 1.2 |
| Panama | Hakre, 2013^30^ | 2009-10 | FSWs in Panama (≥50% registered) | Community | 455 | 71.2 | 455 | 0.70 | -- | 95.0^c^ | -- |
| Panama | Hakre, 2013^30^ | 2009-10 | FSWs in Cocle (≥50% registered) | Community | 64 | 84.4 | 64 | 0.0^b^ | -- | 95.0^c^ | -- |
| Panama | Hakre, 2013^30^ | 2009-10 | FSWs in Colon (≥50% registered) | Community | 150 | 76.7 | 150 | 1.30 | -- | 95.0^c^ | -- |
| Panama | Hakre, 2013^30^ | 2009-10 | FSWs in Chiriqui (≥50% registered) | Community | 155 | 72.3 | 155 | 0.0^b^ | -- | 95.0^c^ | -- |
| Panama | Hakre, 2013^30^ | 2009-10 | FSWs in Herrera & Los Santos (≥50% reg.) | Community | 52 | 75.0 | 52 | 0.0^b^ | -- | 95.0^c^ | -- |
| Panama | Hakre, 2013^30^ | 2009-10 | FSWs in Bocas del Toro (≥50% unregistered) | Community | 95 | 77.9 | 95 | 2.10 | -- | 80.0^c^ | -- |
| Panama | Hakre, 2013^30^ | 2009-10 | FSWs in Veraguas (≥50% unregistered) | Community | 28 | 82.1 | 28 | 0.0^b^ | -- | 80.0^c^ | -- |
| Panama | Soto, 2007^25^ | 2001-02 | Brothel & mobile FSWs | Community | 409 | 73.0 | 418 | 0.2 | -- | 94.1 | 5.7 |
| Peru | Caceres, 2006^31^ | 2003-05 | Low income FSWs in 3 cities | Community | 295 | 48.8 | 295 | 0.30 | -- | 62.7 | -- |
| Peru | Carcamo, 2012^32^ | 2002-03 | FSWs in Barranca | Community | 18 | 77.8 | 168 | 0.0^b^ | -- | -- | -- |
| Peru | Carcamo, 2012^32^ | 2002-03 | FSWs in Chimbote | Community | 36 | 88.9 | 199 | 1.0 | -- | -- | -- |
| Peru | Carcamo, 2012^32^ | 2002-03 | FSWs in Chincha and Ica | Community | 15 | 73.3 | 399 | 1.0 | -- | -- | -- |
| Peru | Carcamo, 2012^32^ | 2002-03 | FSWs in Ilo and Pisco | Community | 18 | 44.4 | 348 | 0.3 | -- | -- | -- |
| Peru | Carcamo, 2012^32^ | 2002-03 | FSWs in Piura | Community | 11 | 72.7 | 193 | 2.1 | -- | -- | -- |
| Peru | Carcamo, 2012^32^ | 2002-03 | FSWs in Sullana | Community | 27 | 51.9 | 200 | 0.0^b^ | -- | -- | -- |
| Peru | Carcamo, 2012^32^ | 2002-03 | FSWs in Tacna | Community | 10 | 60.0 | 205 | 0.5 | -- | -- | -- |
| Peru | Carcamo, 2012^32^ | 2002-03 | FSWs in Talara | Community | 12 | 41.7 | 143 | 1.4 | -- | -- | -- |
| Peru | Carcamo, 2012^32^ | 2002-03 | FSWs in Tumbes | Community | 12 | 83.3 | 74 | 2.7 | -- | -- | -- |
| Peru | Carcamo, 2012^32^ | 2002-03 | FSWs in Arequipa | Community | 10 | 40.0 | 201 | 0.5 | -- | -- | -- |
| Peru | Carcamo, 2012^32^ | 2002-03 | FSWs in Ayacucho | Community | 15 | 60.0 | 147 | 0.7 | -- | -- | -- |
| Peru | Carcamo, 2012^32^ | 2002-03 | FSWs in Cajamarca | Community | 12 | 75.0 | 184 | 0.0^b^ | -- | -- | -- |
| Peru | Carcamo, 2012^32^ | 2002-03 | FSWs in Cerro de Pasco | Community | 17 | 17.7 | 199 | 0.0^b^ | -- | -- | -- |
| Peru | Carcamo, 2012^32^ | 2002-03 | FSWs in Cusco | Community | 17 | 58.8 | 208 | 0.0^b^ | -- | -- | -- |
| Peru | Carcamo, 2012^32^ | 2002-03 | FSWs in Huancayo | Community | 10 | 50.0 | 196 | 0.0^b^ | -- | -- | -- |
| Peru | Carcamo, 2012^32^ | 2002-03 | FSWs in Huaraz | Community | 11 | 72.7 | 140 | 0.7 | -- | -- | -- |
| Peru | Carcamo, 2012^32^ | 2002-03 | FSWs in Juliaca | Community | 11 | 9.1 | 197 | 0.0^b^ | -- | -- | -- |
| Peru | Carcamo, 2012^32^ | 2002-03 | FSWs in Puno | Community | 14 | 28.6 | 201 | 0.0^b^ | -- | -- | -- |
| Peru | Carcamo, 2012^32^ | 2002-03 | FSWs in Huanuco | Community | 21 | 76.2 | 202 | 0.5 | -- | -- | -- |
| Peru | Carcamo, 2012^32^ | 2002-03 | FSWs in Iquitos | Community | 26 | 100.0 | 200 | 1.5 | -- | -- | -- |
| Peru | Carcamo, 2012^32^ | 2002-03 | FSWs in Pucallpa | Community | 32 | 96.9 | 200 | 1.5 | -- | -- | -- |
| Peru | Carcamo, 2012^32^ | 2002-03 | FSWs in Tarapoto | Community | 26 | 88.5 | 159 | 1.9 | -- | -- | -- |
| Peru | Golenbock, 1988^33^ | 1986 | FSWs in Callao | Community | 140 | 91.0 | 140 | 0.0^b^ | -- | 1.4 | -- |
| Peru | Gotuzzo, 1994^34^ | 1991-92 | FSWs at governmental health clinic | STI clinic | 399 | 82.20 | 400 | 0.8 | -- | 54.1 | -- |
| Peru | Perla, 2012^35^ | 2002-03 | Clandestine FSWs in Lima | Community | 211 | 80.10 | 211 | 2.4 | -- | 73.0 | -- |
| Peru | Sanchez, 1998^36^ | 1991-92 | Registered FSWs attending an STI clinic | STI clinic | 283 | 82.0 | 284 | 0.7 | -- | 77.0 | -- |
| Peru | Sanchez, 1998^36^ | 1991-92 | Unregistered FSWs attending an STI clinic | STI clinic | 116 | 82.8 | 116 | 0.9 | -- | 81.4 | -- |
| USA | Cohan, 2005^37^ | 1996-98 | Women with sex work history in California | Community | 226 | 72.9 | 226 | 0.3 | -- | -- | 19.7^d^ |
| USA | Jones, 1998^38^ | 1991-92 | FSWs who are cocaine users (non-injecting) | Community | 303 | 73.4 | 303 | 25.4 | -- | 46.0^c^ | -- |
| USA | Jones, 1998^38^ | 1991-92 | FSWs who are cocaine users (injecting) | Community | 34 | 65.4 | 34 | 23.5 | -- | 46.0^c^ | -- |
| USA | Lutnick, 2008^39^ | -- | FSWs in San Francisco | Community | 250 | 82.0 | 250 | 4.1 | -- | 48.6 | 51.6 |
| **EURO (n=6)** |  |  |  |  |  |  |  |  |  |  |  |
| Greece | Papadogeorgaki, 2006^40^ | 2005 | Greek FSWs | Health center | 240 | 74.6 | 240 | 0.0^b^ | -- | -- | -- |
| Greece | Papadogeorgaki, 2006^40^ | 2005 | Non-Greek FSWs | Health center | 59 | 49.2 | 59 | 0.0^b^ | -- | -- | -- |
| Israel | Linhart, 2008^41^ | -- | Brothel-based FSWs | Brothel | 300 | 60.0 | 300 | 0.3 | -- | 90.70 | -- |
| Russia | Khromova, 2002^42^ | -- | Juvenile and homeless detainee FSWs | Prison | 400 | 29.2 | 400 | 2.8 | -- | -- | -- |
| Slovakia | Bystricka, 2003^43^ | -- | FSWs attending a health center in Bratislava | Health center | 18 | 50.0 | 18 | 5.6 | -- | -- | -- |
| Turkey | Gul, 2008^44^ | 2005 | Brothel-based FSWs in Ankara | Brothel | 130 | 80.0 | 130 | 0.0^b^ | -- | 70.0 | 0.0 |
| **EMRO (n=4)** |  |  |  |  |  |  |  |  |  |  |  |
| Pakistan | Hawkes, 2009^45^ | 2007 | FSWs in Rawalpindi | Community | 426 | 8.0 | 426 | 0.0^b^ | -- | 38.0^c^ | 3.0^e^ |
| Pakistan | Hawkes, 2009^45^ | 2007 | FSWs in Abbottabad | Community | 107 | 4.7 | 107 | 0.0^b^ | -- | 38.0^c^ | 3.0^e^ |
| Tunisia | Znazen, 2010^46^ | 2007 | FSWs engaged in sex work for <5years | Health center | 63 | 47.6 | 63 | 0.0^b^ | -- | 73.0 | -- |
| Tunisia | Znazen, 2010^46^ | 2007 | FSWs engaged in sex work for ≥5years | Health center | 120 | 59.2 | 125 | 0.0^b^ | -- | 54.4 | -- |
| **SEARO (n=71)** | |  |  |  |  |  |  |  |  |  |  |
| Bangladesh | Qutub, 2003^47^ | -- | Brothel-based FSWs in Bangladesh | Brothel | 463 | 94.6 | 463 | 0.0^b^ | -- | 0.0 | -- |
| East Timor | Pisani, 2006^48^ | 2003 | East Timorese & Indonesian FSWs in Dili | Community | 98 | 60.2 | 100 | 3.0 | -- | 36.0 | -- |
| India | Mishra, 2009^49^ | 2004 | FSWs in Mysore, Karnataka | Community | 393 | 64.4 | 393 | 25.2 | -- | -- | -- |
| India | National Rep., 2011^50^ | 2006 | FSWs in Chittoor, Round 1 | Community | 40 | 80.0 | 401 | 8.0 | -- | 85.0 | -- |
| India | National Rep., 2011^50^ | 2009 | FSWs in Chittoor, Round 2 | Community | 40 | 52.5 | 398 | 10.5 | -- | 99.0 | -- |
| India | National Rep., 2011^50^ | 2006 | FSWs in East Godavari, Round 1 | Community | 42 | 81.4 | 422 | 26.3 | -- | 93.0 | -- |
| India | National Rep., 2011^50^ | 2009 | FSWs in East Godavari, Round 2 | Community | 40 | 78.0 | 401 | 23.3 | -- | 99.0 | -- |
| India | National Rep., 2011^50^ | 2006 | FSWs in Guntur, Round 1 | Community | 41 | 82.9 | 405 | 21.3 | -- | 95.0 | -- |
| India | National Rep., 2011^50^ | 2009 | FSWs in Guntur, Round 2 | Community | 41 | 70.7 | 405 | 8.4 | -- | 100.0 | -- |
| India | National Rep., 2011^50^ | 2006 | FSWs in Hyderabad, Round 1 | Community | 40 | 77.5 | 399 | 14.3 | -- | 95.0 | -- |
| India | National Rep., 2011^50^ | 2009 | FSWs in Hyderabad, Round 2 | Community | 40 | 87.8 | 401 | 9.6 | -- | 96.0 | -- |
| India | National Rep., 2011^50^ | 2005 | FSWs in Karimnagar, Round 1 | Community | 41 | 65.1 | 412 | 21.1 | -- | 91.0 | -- |
| India | National Rep., 2011^50^ | 2009 | FSWs in Karimnagar, Round 2 | Community | 40 | 65.9 | 402 | 6.5 | -- | 95.0 | -- |
| India | National Rep., 2011^50^ | 2006 | FSWs in Prakasham, Round 1 | Community | 40 | 53.7 | 404 | 11.1 | -- | 81.0 | -- |
| India | National Rep., 2011^50^ | 2009 | FSWs in Prakasham, Round 2 | Community | 41 | 61.0 | 408 | 13.4 | -- | 96.0 | -- |
| India | National Rep., 2011^50^ | 2006 | FSWs in Visakhapatnam, Round 1 | Community | 41 | 57.1 | 411 | 14.2 | -- | 94.0 | -- |
| India | National Rep., 2011^50^ | 2009 | FSWs in Visakhapatnam, Round 2 | Community | 41 | 58.5 | 409 | 18.2 | -- | 97.0 | -- |
| India | National Rep., 2011^50^ | 2006 | FSWs in Warangal, Round 1 | Community | 42 | 61.9 | 417 | 10.8 | -- | 89.0 | -- |
| India | National Rep., 2011^50^ | 2009 | FSWs in Warangal, Round 2 | Community | 40 | 39.0 | 401 | 15.0 | -- | 99.0 | -- |
| India | National Rep., 2011^50^ | 2006 | FSWs in Bangalore, Round 1 | Community | 67 | 68.6 | 673 | 12.7 | -- | 92.0 | -- |
| India | National Rep., 2011^50^ | 2005 | FSWs in Belgaum, Round 1 | Community | 36 | 83.8 | 360 | 33.9 | -- | 96.0 | -- |
| India | National Rep., 2011^50^ | 2005 | FSWs in Bellary, Round 1 | Community | 42 | 70.8 | 420 | 15.7 | -- | 83.0 | -- |
| India | National Rep., 2011^50^ | 2005 | FSWs in Shimoga, Round 1 | Community | 39 | 59.7 | 390 | 9.7 | -- | 75.0 | -- |
| India | National Rep., 2011^50^ | 2006 | FSWs in Kolhapur, Round 1 | Community | 12 | 83.3 | 115 | 33.0 | -- | 88.0 | -- |
| India | National Rep., 2011^50^ | 2009 | FSWs in Kolhapur, Round 2 | Community | 19 | 75.0 | 190 | 27.4 | -- | 100.0 | -- |
| India | National Rep., 2011^50^ | 2006 | FSWs bar girls in Mumbai, Round 1 | Community | 34 | 50.0 | 338 | 5.9 | -- | 93.0 | -- |
| India | National Rep., 2011^50^ | 2009 | FSWs bar girls in Mumbai, Round 2 | Community | 41 | 63.0 | 405 | 3.1 | -- | 96.3 | -- |
| India | National Rep., 2011^50^ | 2006 | Brothel-based FSWs in Mumbai, Round 1 | Community | 41 | 87.8 | 407 | 28.1 | -- | 97.0 | -- |
| India | National Rep., 2011^50^ | 2009 | Brothel-based FSWs in Mumbai, Round 2 | Community | 40 | 86.6 | 395 | 34.9 | -- | 100.0 | -- |
| India | National Rep., 2011^50^ | 2006 | Street-based FSWs in Mumbai, Round 1 | Community | 39 | 70.2 | 394 | 19.2 | -- | 97.0 | -- |
| India | National Rep., 2011^50^ | 2009 | Street-based FSWs in Mumbai, Round 2 | Community | 39 | 85.0 | 385 | 32.3 | -- | 100.0 | -- |
| India | National Rep., 2011^50^ | 2006 | FSWs in Parbhani, Round 1 | Community | 37 | 52.2 | 367 | 16.1 | -- | 93.0 | -- |
| India | National Rep., 2011^50^ | 2009 | FSWs in Parbhani, Round 2 | Community | 30 | 80.6 | 303 | 14.9 | -- | 99.0 | -- |
| India | National Rep., 2011^50^ | 2006 | Brothel-based FSWs in Pune, Round 1 | Community | 40 | 80.9 | 404 | 38.7 | -- | 98.0 | -- |
| India | National Rep., 2011^50^ | 2009 | Brothel-based FSWs in Pune, Round 2 | Community | 40 | 65.8 | 403 | 20.3 | -- | 100.0 | -- |
| India | National Rep., 2011^50^ | 2006 | Non-brothel-based FSWs in Pune, Round 1 | Community | 26 | 96.2 | 257 | 37.0 | -- | 97.0 | -- |
| India | National Rep., 2011^50^ | 2009 | Non-brothel-based FSWs in Pune, Round 2 | Community | 27 | 88.9 | 266 | 21.8 | -- | 98.0 | -- |
| India | National Rep., 2011^50^ | 2006 | Brothel-based FSWs in Thane, Round 1 | Community | 40 | 35.9 | 401 | 18.6 | -- | 99.0 | -- |
| India | National Rep., 2011^50^ | 2009 | Brothel-based FSWs in Thane, Round 2 | Community | 38 | 81.5 | 384 | 33.1 | -- | 100.0 | -- |
| India | National Rep., 2011^50^ | 2006 | Street-based FSWs in Thane, Round 1 | Community | 39 | 58.3 | 394 | 7.0 | -- | 98.0 | -- |
| India | National Rep., 2011^50^ | 2009 | Street-based FSWs in Thane, Round 2 | Community | 40 | 74.4 | 395 | 11.8 | -- | 99.0 | -- |
| India | National Rep., 2011^50^ | 2006 | FSWs in Yevatmal, Round 1 | Community | 15 | 100.0 | 153 | 37.3 | -- | 96.0 | -- |
| India | National Rep., 2011^50^ | 2009 | FSWs in Yevatmal, Round 2 | Community | 16 | 87.5 | 157 | 26.8 | -- | 99.0 | -- |
| India | National Rep., 2011^50^ | 2006 | FSWs in Chennai, Round 1 | Community | 41 | 31.7 | 410 | 2.2 | -- | 96.0 | -- |
| India | National Rep., 2011^50^ | 2009 | FSWs in Chennai, Round 2 | Community | 40 | 37.5 | 397 | 2.4 | -- | 99.0 | -- |
| India | National Rep., 2011^50^ | 2006 | FSWs in Coimbatore, Round 1 | Community | 41 | 56.1 | 410 | 6.3 | -- | 93.0 | -- |
| India | National Rep., 2011^50^ | 2009 | FSWs in Coimbatore, Round 2 | Community | 40 | 58.9 | 400 | 6.3 | -- | 99.0 | -- |
| India | National Rep., 2011^50^ | 2006 | FSWs in Dharmapuri, Round 1 | Community | 41 | 75.6 | 408 | 12.4 | -- | 95.0 | -- |
| India | National Rep., 2011^50^ | 2009 | FSWs in Dharmapuri , Round 2 | Community | 41 | 48.2 | 406 | 8.8 | -- | 91.0 | -- |
| India | National Rep., 2011^50^ | 2006 | FSWs in Madurai, Round 1 | Community | 40 | 48.8 | 402 | 4.3 | -- | 84.0 | -- |
| India | National Rep., 2011^50^ | 2009 | FSWs in Madurai, Round 2 | Community | 40 | 58.2 | 396 | 8.3 | -- | 100.0 | -- |
| India | National Rep., 2011^50^ | 2006 | FSWs in Salem, Round 1 | Community | 40 | 72.5 | 402 | 12.5 | -- | 93.0 | -- |
| India | National Rep., 2011^50^ | 2009 | FSWs in Salem, Round 2 | Community | 41 | 53.6 | 407 | 6.7 | -- | 99.0 | -- |
| India | National Rep., 2011^50^ | 2006 | FSWs in Dimapur, Round 1 | Community | 43 | 52.6 | 426 | 11.6 | -- | 36.0 | -- |
| India | National Rep., 2011^50^ | 2009 | FSWs in Dimapur, Round 2 | Community | 42 | 44.7 | 417 | 11.4 | -- | 72.0 | -- |
| India | Sarna, 2013^51^ | 2010 | FSWs in Nellore | Community | 529 | 60.7 | 529 | 5.3 | -- | 47.2 | -- |
| India | Shahmanesh, 2009^52^ | 2004-05 | FSWs in Goa | Community | 326 | 57.2 | 326 | 25.7 | -- | 74.4 | -- |
| India | Uma, 2005^53^ | 2004 | FSWs bacterial vaginosis positive | Community | 260 | 73.5 | 260 | 5.3 | -- | -- | -- |
| India | Uma, 2005^53^ | 2004 | FSWs bacterial vaginosis intermediate | Community | 92 | 67.4 | 92 | 11.0 | -- | -- | -- |
| India | Uma, 2005^53^ | 2004 | FSWs bacterial vaginosis negative | Community | 230 | 56.1 | 230 | 1.3 | -- | -- | -- |
| Indonesia | Davies, 2007^54^ | 1999-00 | FSWs in Kupang | STI clinic | 176 | 86.9 | 176 | 0.0^b^ | -- | 4.0 | -- |
| Thailand | Limpakarnjanarat, 1999^55^ | 1991-94 | Brothel-based FSWs at Chiang province | STI clinic | 280 | 78.2 | 280 | 47.1 | -- | 32.8^c^ | -- |
| Thailand | Limpakarnjanarat, 1999^55^ | 1991-94 | Non-brothel-based FSWs at Chiang province | STI clinic | 220 | 72.3 | 220 | 12.7 | -- | 32.8^c^ | -- |
| Vietnam | Vu Thuong, 2007^56^ | 2002 | FSWs in Lai Chau | Community | 100 | 5.0 | 100 | 2.0 | -- | 45.3^c^ | 3.9^de^ |
| Vietnam | Vu Thuong, 2007^56^ | 2002 | FSWs in Quang Tri | Community | 101 | 20.8 | 101 | 1.0 | -- | 45.3^c^ | 3.9^de^ |
| Vietnam | Vu Thuong, 2007^56^ | 2002 | FSWs in Dong Thap | Community | 149 | 32.2 | 149 | 4.7 | -- | 45.3^c^ | 3.9^de^ |
| Vietnam | Vu Thuong, 2007^56^ | 2002 | FSWs in An Giang | Community | 300 | 33.3 | 300 | 7.0 | -- | 45.3^c^ | 3.9^de^ |
| Vietnam | Vu Thuong, 2007^56^ | 2002 | FSWs in Kien Giang | Community | 253 | 30.0 | 253 | 4.0 | -- | 45.3^c^ | 3.9^de^ |
| Vietnam | Vu Thuong, 2007^56^ | 2004 | FSWs in Lai Chau | Community | 99 | 20.2 | 99 | 2.0 | -- | 52.8^c^ | 3.1^d^ |
| Vietnam | Vu Thuong, 2007^56^ | 2004 | FSWs in Quang Tri | Community | 100 | 33.0 | 100 | 1.0 | -- | 52.8^c^ | 2.0^d^ |
| Vietnam | Vu Thuong, 2007^56^ | 2004 | FSWs in Dong Thap | Community | 199 | 25.1 | 199 | 2.6 | -- | 52.8^c^ | 0.0^d^ |
| Vietnam | Vu Thuong, 2007^56^ | 2004 | FSWs in An Giang | Community | 285 | 23.5 | 285 | 5.3 | -- | 52.8^c^ | 2.1^d^ |
| Vietnam | Vu Thuong, 2007^56^ | 2004 | FSWs in Kien Giang | Community | 298 | 24.2 | 298 | 4.1 | -- | 52.8^c^ | 2.7^d^ |
| **WPRO (n=49)** | |  |  |  |  |  |  |  |  |  |  |
| Cambodia | Saphonn, 2006^57^ | 2000-02 | FSWs first-time STI clinic attendees | STI clinic | 938 | 38.8 | 938 | 27.4 | -- | -- | -- |
| China | Chen, 1998^58^ | 1993-94 | FSWs in massage parlors in Taiwan | Mass. parlors | 206 | 2.9 | 287 | 0.0^b^ | -- | 94.0^c^ | -- |
| China | Chen, 1998^58^ | 1994-96 | FSWs in massage parlors in Taiwan | Mass. parlors | 81 | 1.2 | 242 | 0.0^b^ | -- | 94.0^c^ | -- |
| China | Chen, 1998^58^ | 1993-94 | FSWs in karaoke bars in Taiwan | Karaoke bars | 557 | 7.5 | 557 | 0.4 | -- | -- | -- |
| China | Chen, 1998^58^ | 1993-94 | Brothel-based FSWs in Taiwan | Brothel | 159 | 1.3 | 159 | 0.0^b^ | -- | 45.2^c^ | -- |
| China | Chen, 1998^58^ | 1994-96 | Brothel-based FSWs in Taiwan | Brothel | 142 | 4.9 | 156 | 0.0^b^ | -- | 45.2^c^ | -- |
| China | Chen, 2005^59^ | 1999-00 | FSWs aged 15-19 years in Kunming | STI clinic | 70 | 4.3 | 70 | 84.3 | -- | 45.2^c^ | -- |
| China | Chen, 2005^59^ | 1999-00 | FSWs aged 20-24 years in Kunming | STI clinic | 204 | 9.8 | 204 | 86.8 | -- | 45.2^c^ | -- |
| China | Chen, 2005^59^ | 1999-00 | FSWs aged 25-29 years in Kunming | STI clinic | 144 | 13.2 | 144 | 79.9 | -- | 45.2^c^ | -- |
| China | Chen, 2005^59^ | 1999-00 | FSWs aged 30-34 years in Kunming | STI clinic | 62 | 9.7 | 62 | 85.5 | -- | 45.2^c^ | -- |
| China | Chen, 2005^59^ | 1999-00 | FSWs aged 35-39 years in Kunming | STI clinic | 25 | 16.0 | 25 | 88.0 | -- | 45.2^c^ | -- |
| China | Chen, 2013^60^ | 2009 | FSWs in Wuzhou and Hezhou in Guangxi | Community | 2453 | 54.9 | 2,453 | 0.7 | -- | 79.2 | -- |
| China | Fu, 2014^61^ | -- | Low fee FSWs in Guangdong | Community | 196 | 57.1 | 196 | 1.0 | -- | 21.1 | -- |
| China | Fu, 2014^61^ | -- | Medium fee FSWs in Guangdong | Community | 379 | 16.9 | 379 | 0.0^b^ | -- | 9.6 | -- |
| China | Han, 2016^62^ | 2012 | Low fee FSWs | Community | 417 | 31.7 | 417 | 0.7 | -- | 42.3 | 4.8 |
| China | Han, 2016^62^ | 2012 | Medium fee FSWs | Community | 1,070 | 26.4 | 1,070 | 0.3 | -- | 55.5 | 1.3 |
| China | Jing, 2017^63^ | 1994 | Vietnamese FSWs in Hekou (June 2014) | Community | 219 | 57.1 | 219 | 3.2 | -- | -- | -- |
| China | Jing, 2017^63^ | 1994 | Vietnamese FSWs in Hekou (Dec 2014) | Community | 245 | 58.4 | 245 | 2.0 | -- | -- | -- |
| China | Jing, 2017^63^ | 1995 | Vietnamese FSWs in Hekou (May 2015) | Community | 265 | 38.1 | 265 | 1.9 | -- | -- | -- |
| China | Jing, 2017^63^ | 1995 | Vietnamese FSWs in Hekou (Nov 2015) | Community | 329 | 51.1 | 329 | 1.8 | -- | -- | -- |
| China | Li, 2014^64^ | 2013 | FSWs from multiple venues | Community | 460 | 43.0 | 460 | 0.2 | -- | -- | -- |
| China | Luo, 2015^65^ | 2012 | FSWs not using vaginal douching in Yunnan | Community | 134 | 56.0 | 134 | 5.2 | -- | 71.9 | 6.7 |
| China | Luo, 2015^65^ | 2012 | FSWs using vaginal douching in Yunnan | Community | 699 | 70.8 | 699 | 11.0 | -- | 78.9 | 9.6 |
| China | Ngo, 2008^66^ | 2004 | FSWs in Kunming | STI clinic | 310 | 45.2 | 310 | 3.9 | -- | 11.6 | -- |
| China | Remis, 2010^67^ | 2009 | FSWs in Shanghai | Community | 750 | 3.1 | 750 | 0.1 | -- | -- | -- |
| China | Wang, 2006^68^ | 2005 | FSWs in a mining township | Community | 327 | 63.7 | 237 | 20.7 | -- | -- | -- |
| China | Wang, 2012^69^ | 2006 | FSWs in Kaiyuan (Fall 2006) | Community | 741 | 67.3 | 741 | 10.2 | -- | -- | -- |
| China | Wang, 2012^69^ | 2006 | FSWs from Kaiyuan (Spring 2006) | Community | 748 | 67.9 | 748 | 11.9 | -- | -- | -- |
| China | Wang, 2012^69^ | 2007 | FSWs from Kaiyuan (Fall 2007) | Community | 705 | 70.8 | 705 | 13.1 | -- | -- | -- |
| China | Wang, 2012^69^ | 2007 | FSWs from Kaiyuan (Spring 2007) | Community | 440 | 62.7 | 440 | 11.4 | -- | -- | -- |
| China | Wang, 2012^69^ | 2008 | FSWs from Kaiyuan (Fall 2008) | Community | 587 | 68.1 | 587 | 11.2 | -- | -- | -- |
| China | Wang, 2012^69^ | 2008 | FSWs from Kaiyuan (Spring 2008) | Community | 558 | 71.2 | 558 | 12.2 | -- | -- | -- |
| China | Wang, 2012^69^ | 2009 | FSWs from Kaiyuan (Fall 2009) | Community | 548 | 71.3 | 548 | 16.2 | -- | -- | -- |
| China | Wang, 2012^69^ | 2009 | FSWs from Kaiyuan (Spring 2009) | Community | 548 | 70.4 | 548 | 15.5 | -- | -- | -- |
| China | Wang, 2015^70^ | 2009 | Vietnamese FSWs in China | Community | 233 | 60.9 | 233 | 7.7 | -- | 90.1 | -- |
| China | Wang, 2015^70^ | 2009 | Chinese FSWs | Community | 112 | 52.7 | 112 | 0.9 | -- | 100.0 | -- |
| China | Wei, 2004^71^ | 1999 | Sex- hospitality girls in Wuhan | Community | 101 | 29.7 | 147 | 0.0^b^ | -- | 51.7 | 8.2 |
| China | Xu, 2008^72^ | 2006 | FSWs from entertainment venues | Community | 96 | 70.8 | 96 | 8.3 | -- | 54.2 | 7.3 |
| China | Xu, 2012^73^ | 2007 | FSWs drug users (Mar-Jul 2007) | Community | 150 | 86.7 | 150 | 43.3 | -- | 84.7 | -- |
| China | Xu, 2012^73^ | 2007 | FSWs non-drug users (Mar-Jul 2007) | Community | 555 | 66.8 | 555 | 4.9 | -- | 86.7 | -- |
| China | Xu, 2013^74^ | 2006-07 | FSWs drug users (Mar 2006-Apr 2007) | Community | 261 | 86.6 | 261 | 39.1 | -- | 84.7 | 7.4^e^ |
| China | Xu, 2013^74^ | 2006-07 | FSWs non-drug users (Mar 2006-Apr 2007) | Community | 1,381 | 66.8 | 1,381 | 4.8 | -- | 86.7 | 7.4^e^ |
| China | Yang, 2011^75^ | 2008 | FSWs in entertainment establishments | Community | 411 | 45.5 | 411 | 0.0^b^ | -- | 78.7 | -- |
| China | Yang, 2011^75^ | 2009 | FSWs in entertainment establishments | Community | 411 | 50.1 | 411 | 0.0^b^ | -- | 82.0 | -- |
| China | Yao, 2012^76^ | 2007 | FSWs drug users (Sep-Oct 2007) | Community | 94 | 92.6 | 94 | 38.3 | -- | -- | 81.9^f^ |
| China | Yao, 2012^76^ | 2007 | FSWs non-drug users (Sep-Oct 2007) | Community | 305 | 59.7 | 305 | 4.0 | -- | -- | -- |
| China | Zhang, 2014^77^ | 2011 | FSWs aged 18-25 years in Shanghai | Community | 336 | 46.4 | 336 | 0.0^b^ | -- | 49.3^c^ | -- |
| China | Zhang, 2014^77^ | 2011 | FSWs aged 26-35 years in Shanghai | Community | 196 | 59.2 | 196 | 0.0^b^ | -- | 49.3^c^ | -- |
| China | Zhang, 2014^77^ | 2011 | FSWs aged ≥36 years in Shanghai | Community | 68 | 60.3 | 68 | 0.0^b^ | -- | 49.3^c^ | -- |

AFRO, African Region; AMRO, Region of the Americas; ART, antiretroviral therapy; Collect, collection; Cov, coverage; Domin Rep, Dominican Republic; EMRO, Eastern Mediterranean Region; EURO, European Region; FSWs, female sex workers; HIV, human immunodeficiency virus; HSV-2, herpes simplex virus type 2; Mass, massage; National Rep, National Report; Prev, prevalence; Prop, proportion; RDS, respondent-driven sampling; Reg, registered; SEARO, South-East Asia Region; STI, sexually transmitted infection; USA, United States of America; WPRO, Western Pacific Region.

^a^Consistent condom use measures were based on self-reported condom use at last sex with client, or alternatively on self-reported “consistent/regular” condom use, or condom use “all the time” during commercial sex acts.

^b^Studies reporting zero HIV prevalence were excluded from subsequent analysis.

^c^Strata were considered to have the same level of consistent condom use as the overall sample.

^d^Proportion of FSWs who reported ever injecting drugs.

^e^Strata were considered to have the same level of injecting drug use as the overall sample.

^f^Proportion of drug-using FSWs who reported injecting drug use.

**Figure S1.** Regional maps illustrating countries’ data contribution in terms of the total number of studies and the total number of FSWs participating in those studies. Map showing data contribution from A) Africa, B) Americas, and C) Other world regions. Maps were created using Tableau Desktop v.10.1^78^.

1. **Africa**


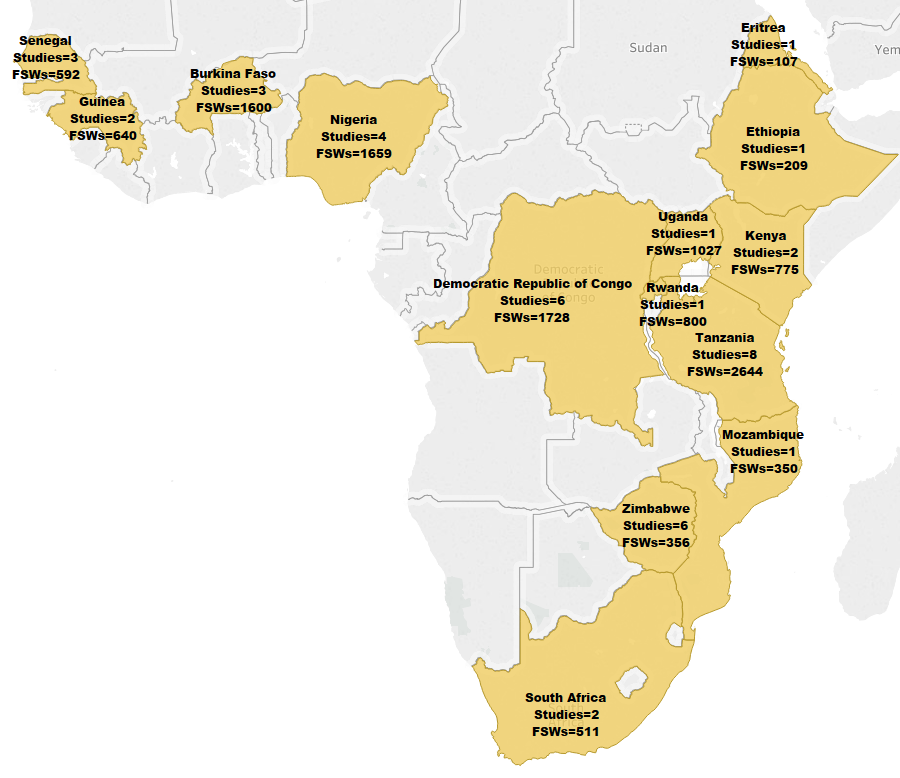


1. **Americas**


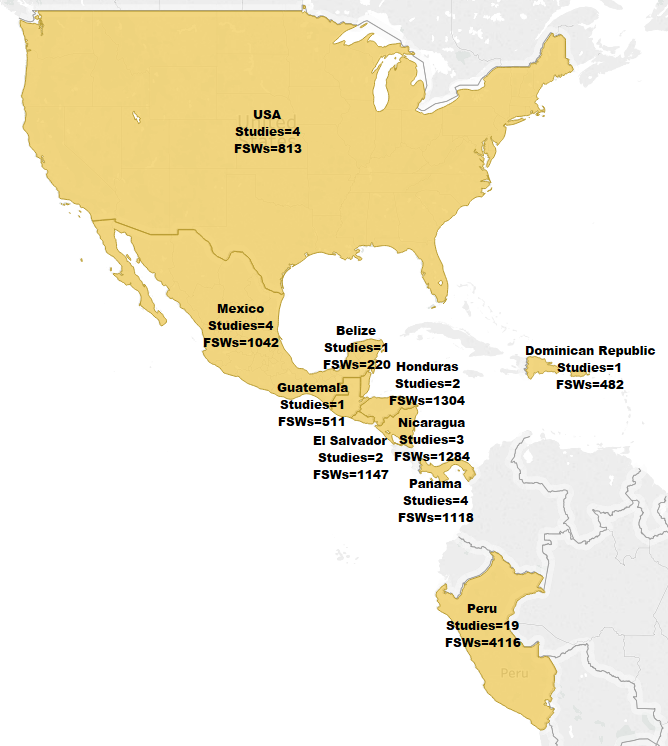


1. **Other world regions**


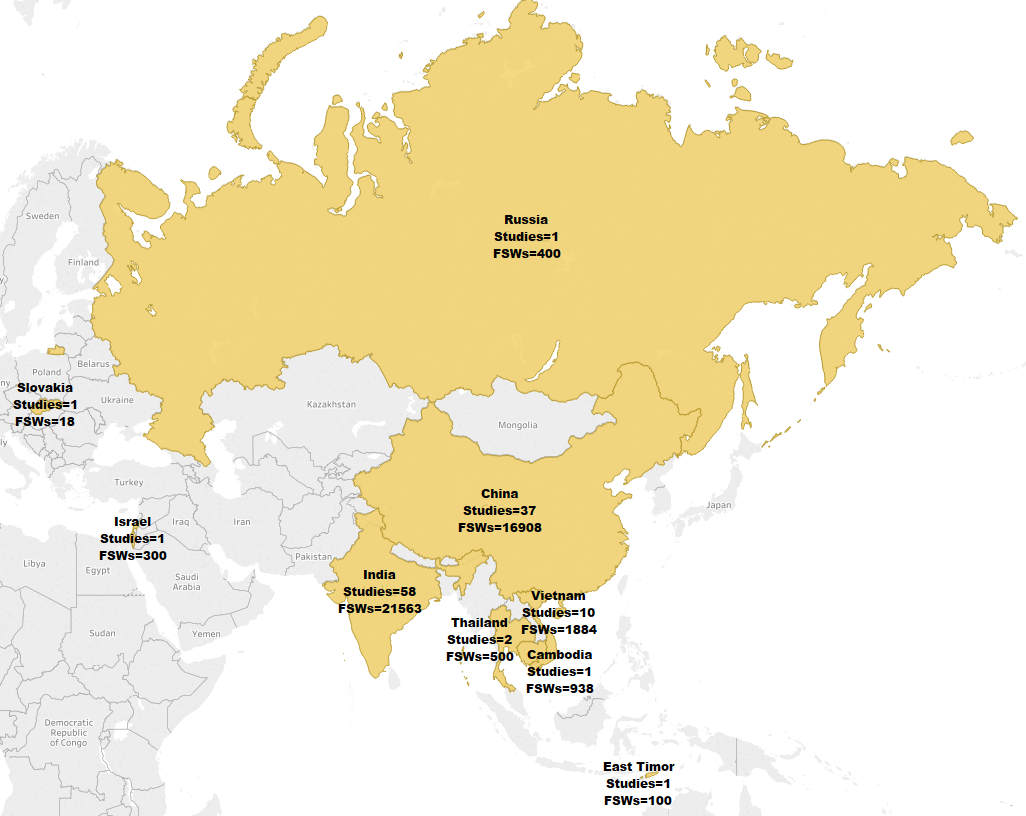


**Figure S2.** Forest plot showing the results of meta-analyses on studies reporting HIV prevalence among female sex workers stratified by HSV-2 prevalence level in A) Africa, B) other world regions, and C) globally. Forest plots were generated using R v.3.4.2^79^.

1. **Africa**


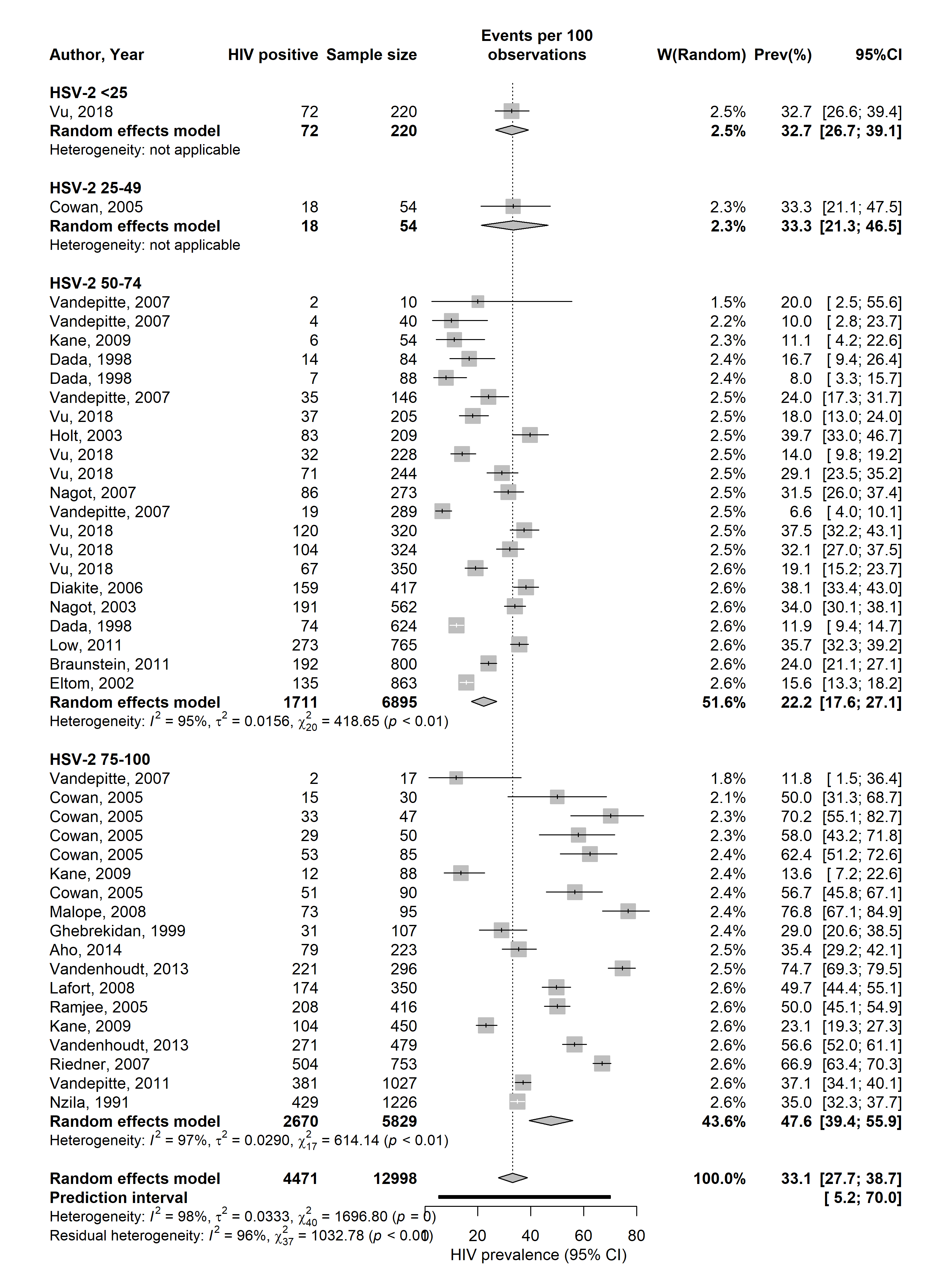


1. **Other world regions**


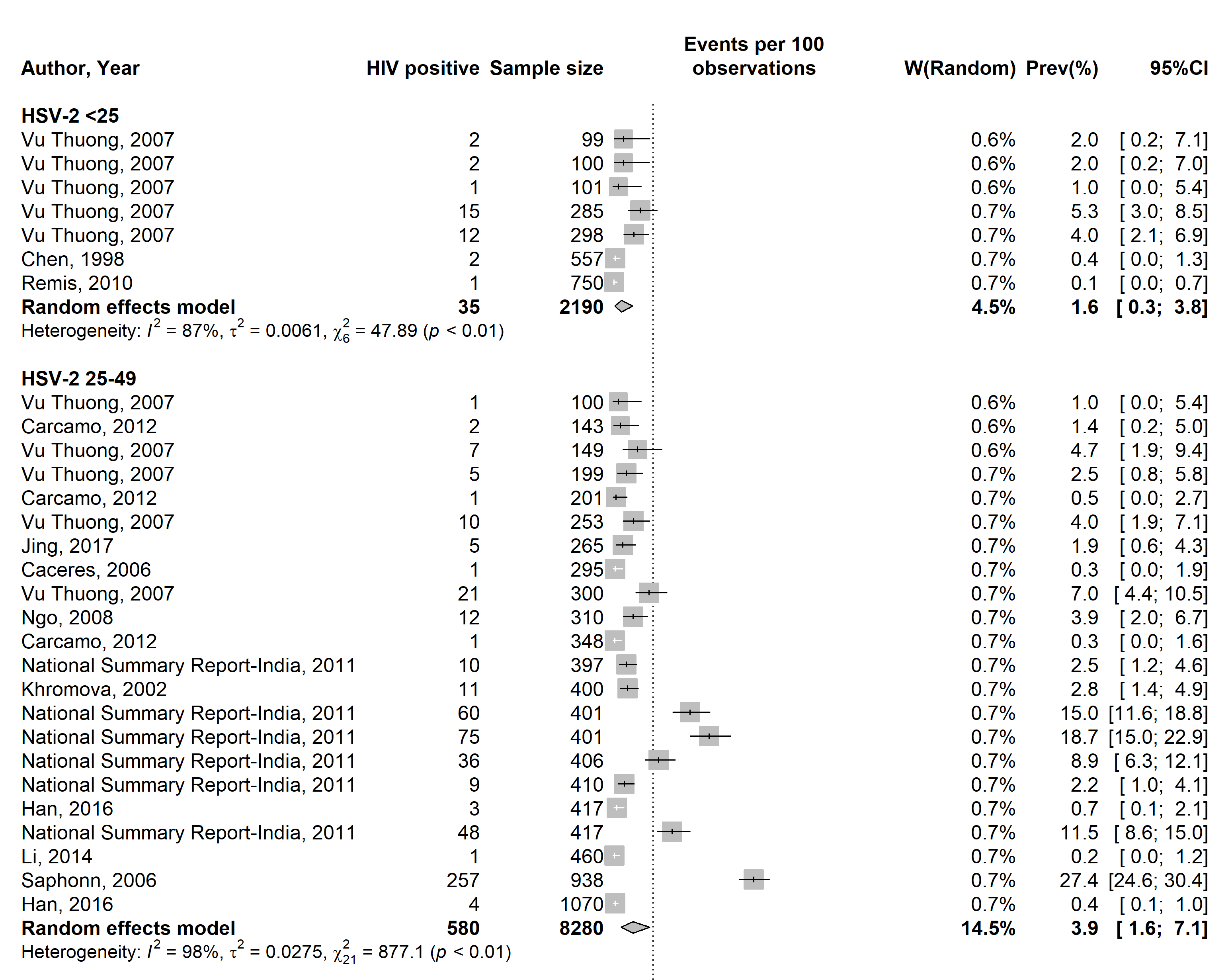

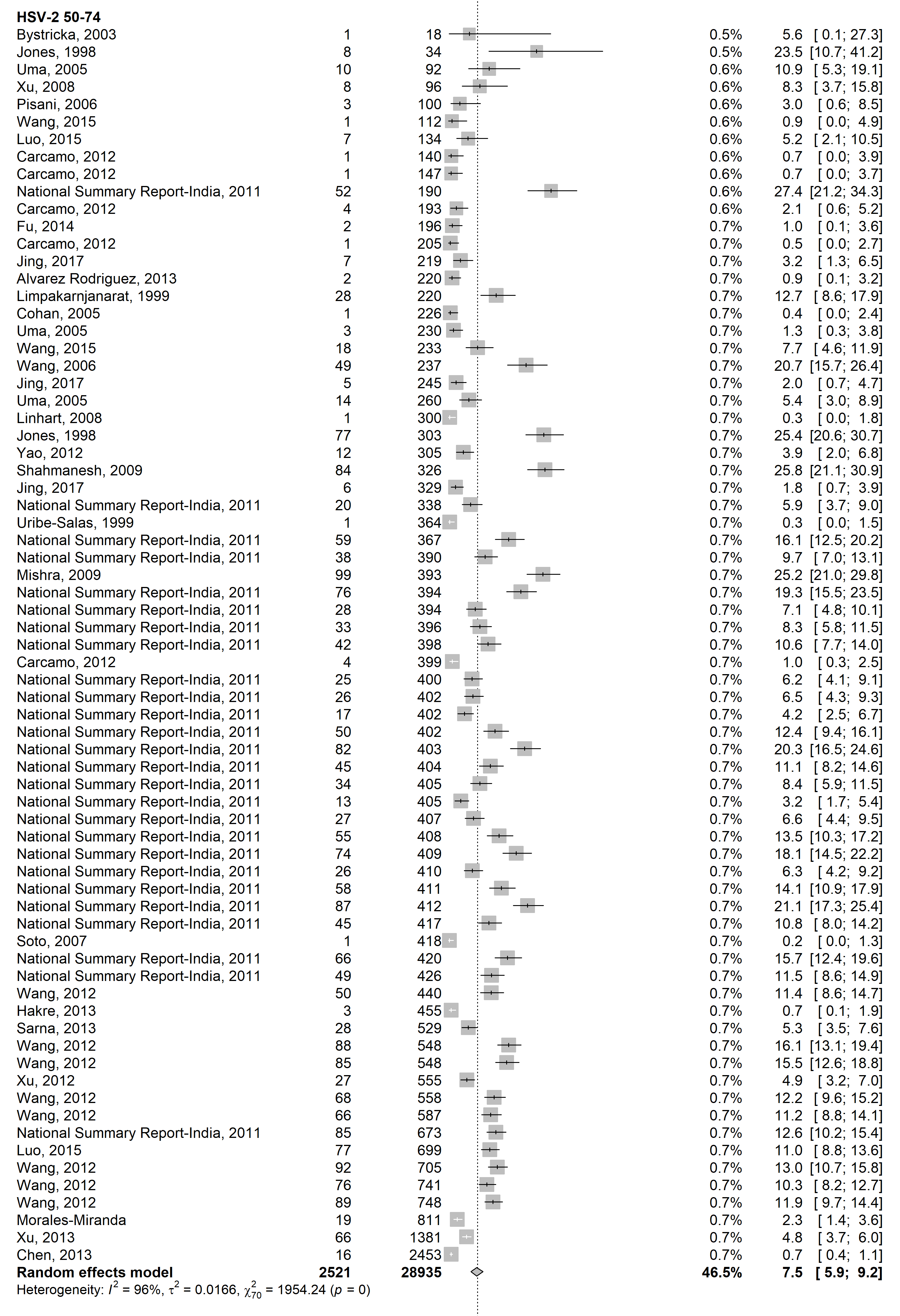

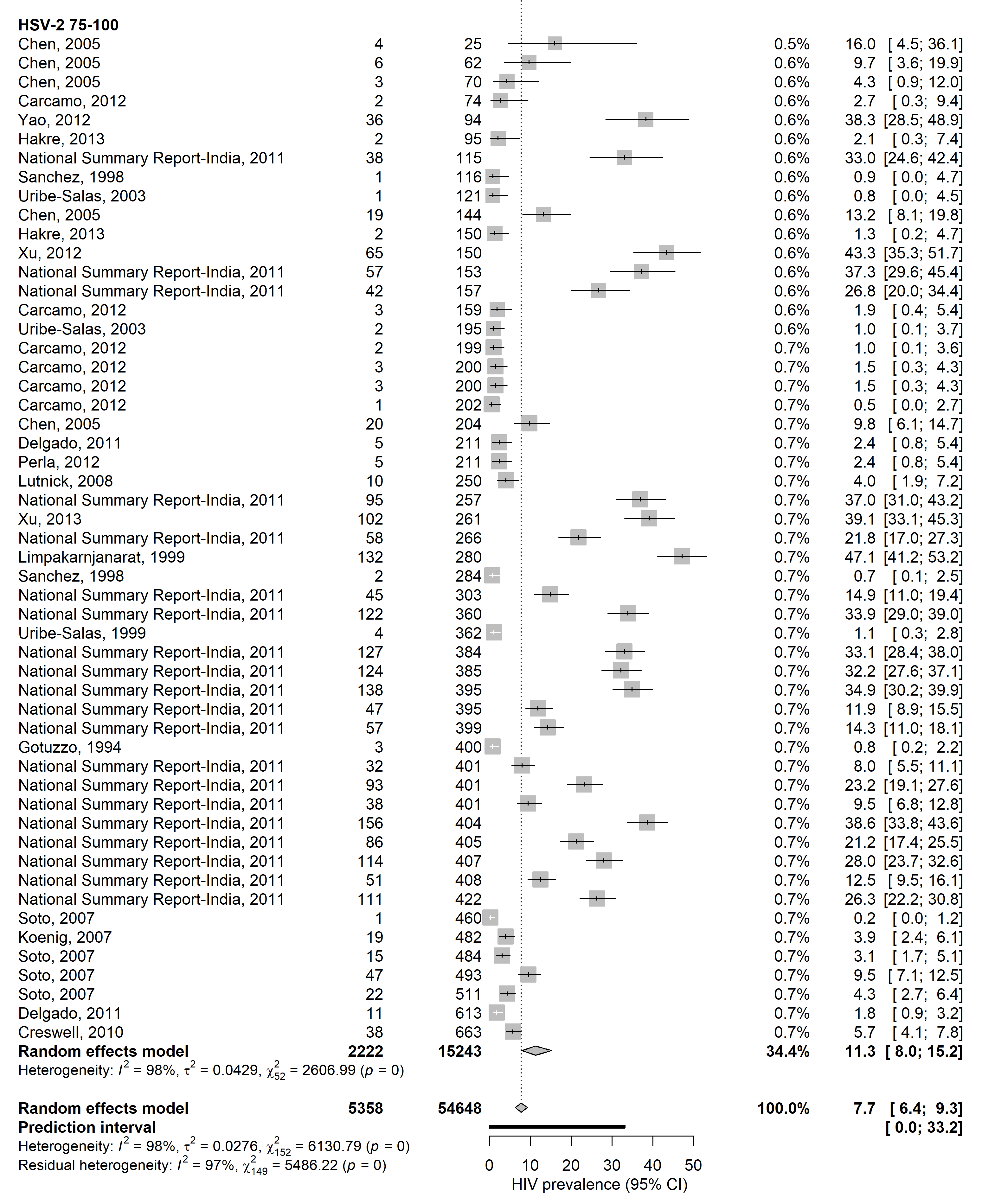


1. **Global**


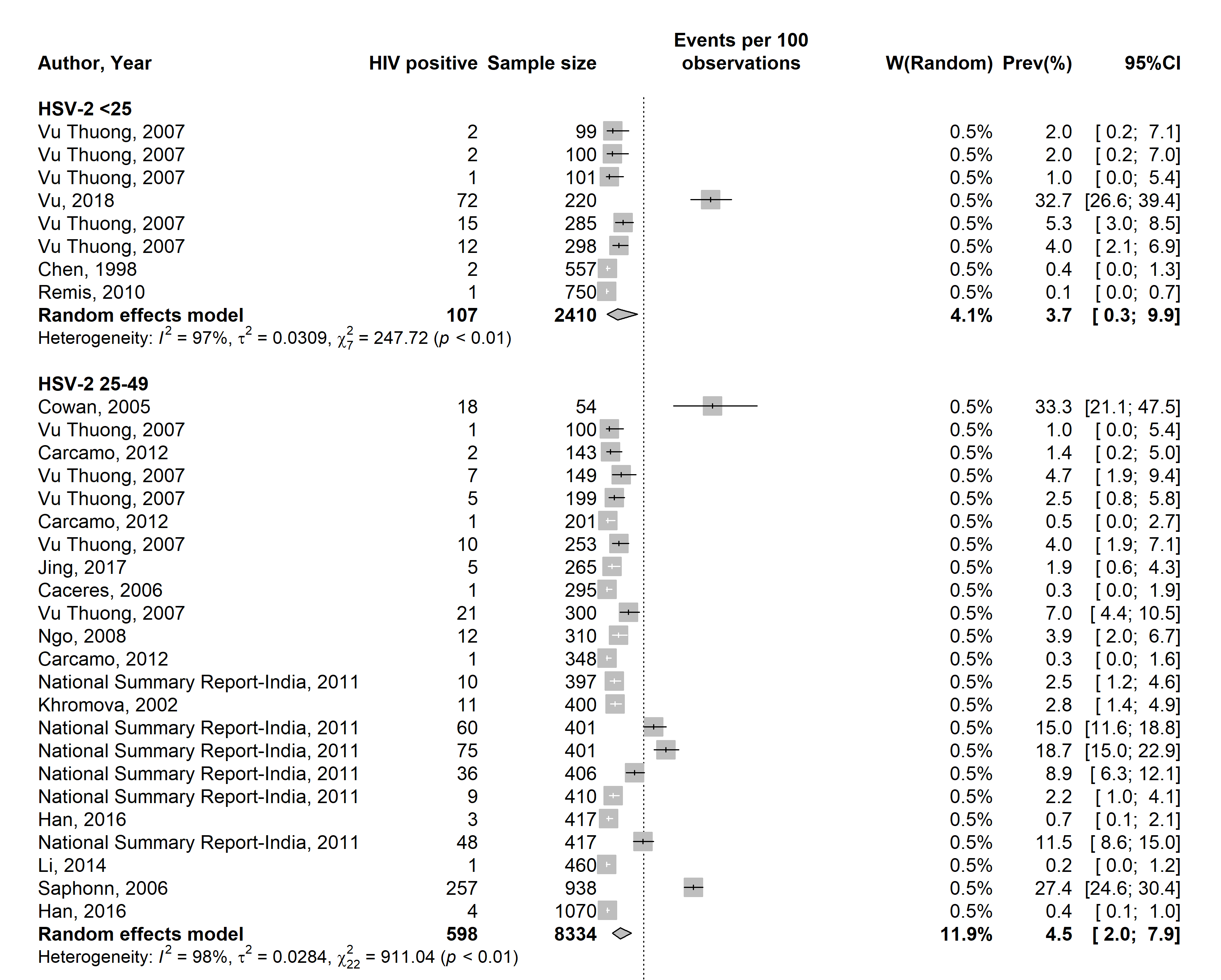

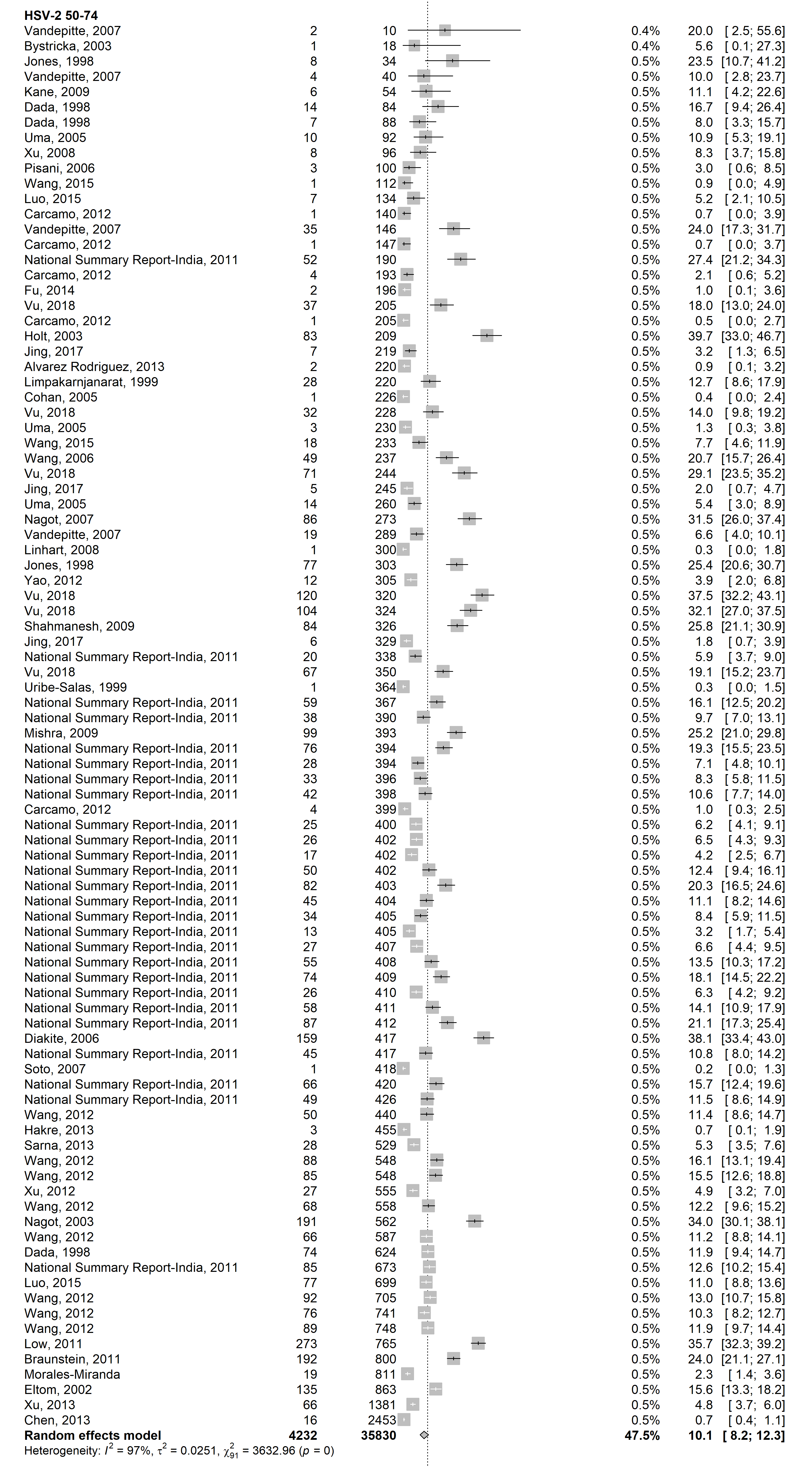

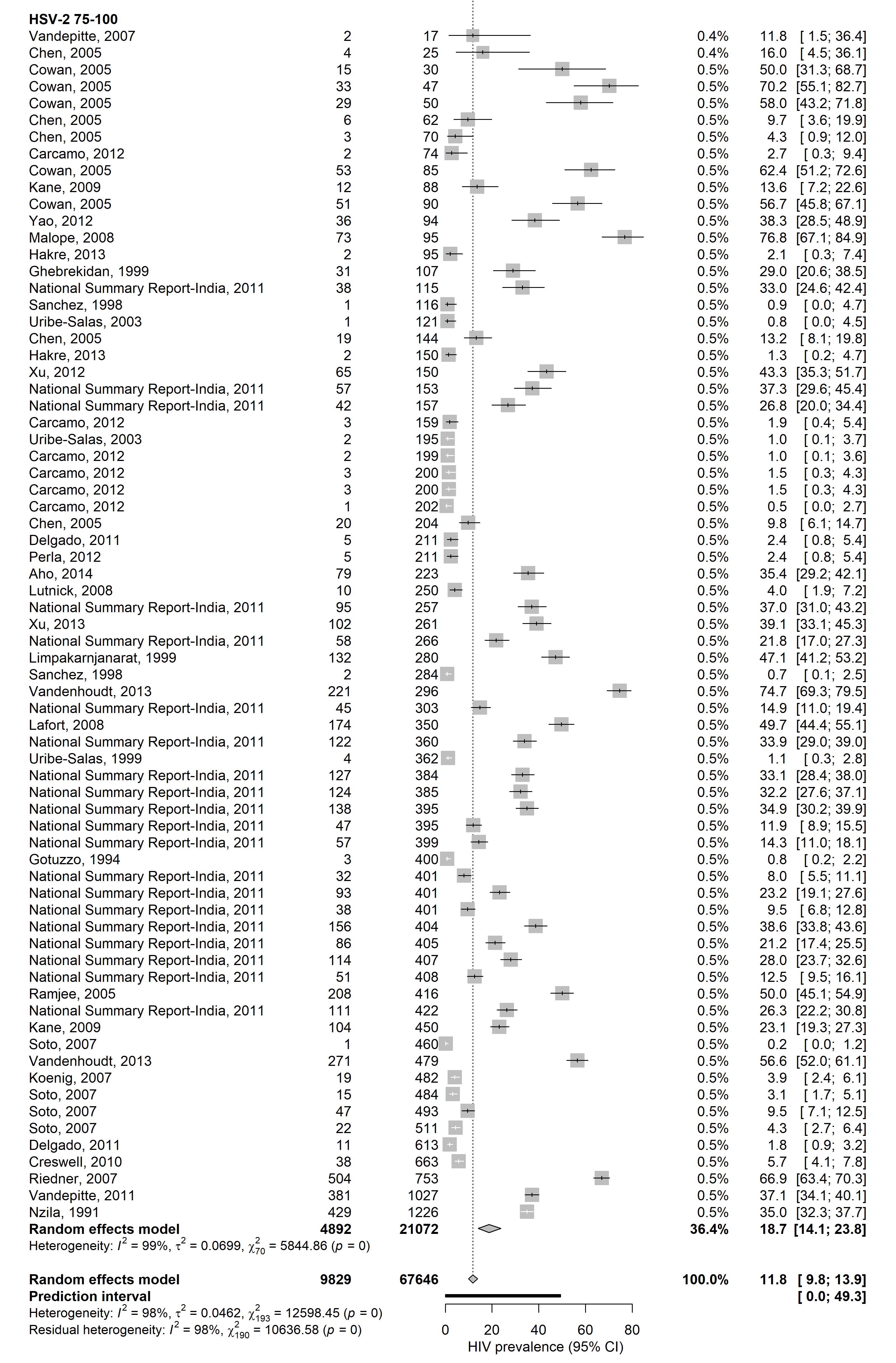


**Box S1.** Search criteria for the systematic review of the global association of herpes simplex virus type 2 (HSV-2) and HIV prevalence measures among female sex workers.

| **PubMed** (September 3rd, 2019) |
| --- |
| ***Sex work*** |
| "Extramarital Relations"[Mesh] OR “Sex Work*”[Mesh] OR "Sex/analysis"[Mesh] OR "Sex/statistics and numerical data"[Mesh] OR "Sexual partners"[Mesh] OR "Sex Trafficking/epidemiology"[Mesh] OR "Sex Trafficking/statistics and numerical data"[Mesh] OR Sex work*[Text] OR Sexual work*[Text] OR Sexwork*[Text] OR Sex-work*[Text] OR Sexual partner*[Text] OR Sex partner*[Text] OR Sexual contact*[Text] OR FSW[Text] OR FSWs[Text] OR CSW[Text] OR CSWs[Text] OR SW[Text] OR SWs[Text] OR TSW[Text] OR TSWs[Text] OR TS[Text] OR Travailleuse* sexe[Text] OR Travailleuse* sex[Text] OR Bar girl*[Text] OR Callgirl*[Text] OR Call girl*[Text] OR Escort*[Text] OR Masseuse*[Text] OR Hostess*[Text] OR ((Premarital[Text] OR Pre-marital[Text] OR Pre marital[Text] OR Extramarital[Text] OR Extra-marital[Text] OR Extra marital[Text] OR Illicit[Text] OR Illegal[Text]) AND (Sex[Text] OR Sexual[Text] OR Relation*[Text])) OR Outside marriage[Text] OR Out of marriage[Text] OR “Illegal social behavior”[Text] OR “Illegal social behaviour”[Text] OR Adultery[Text] OR Prostitut*[Text] OR Promiscu*[Text] OR Female entertain*[Text] OR Sex entertain*[Text] OR Sexual* entertain*[Text] OR Entertainment work*[Text] OR Sex industr*[Text] OR Sex establishment*[Text] OR Brothel*[Text] OR Red light[Text] OR Red-light[Text] OR Red district*[Text] OR Nightclub*[Text] OR Pimp[Text] OR ((Intergenerational[Text] OR Cross-generation*[Text] OR Cross-generational[Text] OR Recreational[Text] OR Commercial[Text] OR Transaction*[Text] OR Casual[Text] OR Group[Text] OR Informal[Text] OR Street[Text] OR Migrant*[Text] OR Survival[Text] OR Occupational[Text] OR Tourism[Text]) AND (Sex[Text] OR Sexual*[Text])) OR Sex seeking[Text] OR Sex-seeking[Text] OR Solicit*[Text] OR ((Provision*[Text] OR Provider*[Text] OR Provid*[Text] OR Sell*[Text] OR Sold[Text] OR Exchang*[Text] OR Trad*[Text] OR Favor*[Text] OR Consum*[Text] OR Commodi*[Text] OR Paid[Text] OR Paying[Text] OR Pay[Text] OR Payer*[Text] OR Buying[Text] OR Buy[Text] OR Buyer*[Text] OR Charg*[Text] OR Engag*[Text] OR Service*[Text] OR Money[Text] OR Cash[Text] OR Drug*[Text] OR Goods[Text] OR Gift*[Text]) AND (Sex[Text] OR Sexual*[Text])) OR Hidden population*[Text] OR Hard to reach population*[Text] OR Hard-to-reach population*[Text] OR Core group*[Text] OR Core risk group*[Text] OR Vulnerable women[Text] OR Vulnerable population*[Text] OR Vulnerable female*[Text] OR Most-at-risk population*[Text] OR Most at risk population*[Text] OR High risk population*[Text] OR High-risk population*[Text] OR Population* at high risk[Text] OR Population* at high-risk[Text] OR ((Traffick*[Text] OR Slave*[Text] OR Coerc*[Text] OR Abduct*[Text] OR Exploit*[Text] OR Abuse*[Text] OR Violence[Text]) AND (Sex[Text] OR Sexual*[Text])) |
| ***Herpes simplex virus-2*** |
| (Simplexvirus[MeSH] OR Herpes Simplex[MeSH] OR Herpes Hominis[Text] OR HSV type-2[Text] OR HSV type 2[Text] OR HSV2[Text] OR HSV-2[Text] OR HSV 2[Text] OR HHV2[Text] OR HHV-2[Text] OR HHV 2[Text] OR Herpes simplex virus type 2[Text] OR Herpes simplex virus type-2[Text] OR herpes simplex virus 2[Text] OR herpes simplex virus-2[Text] OR herpes simplex type 2[Text] OR herpes simplex type-2[Text] OR herpes simplex 2[Text] OR herpes simplex-2[Text] OR Herpesvirus type 2[Text] OR Herpesvirus type-2[Text] OR Herpesvirus 2[Text] OR Herpesvirus-2[Text] OR Herpes virus type 2[Text] OR Herpes virus type-2[Text] OR Herpes virus 2[Text] OR Herpes virus-2[Text] OR genital herpes[Text] OR Human herpes virus[Text] OR Herpes virus[Text] OR Herpes Genitalis[Text] OR Herpes Labialis[Text]) |
| ***HIV*** |
| ("HIV"[Mesh] OR "HIV Seropositivity"[Mesh] OR "HIV Antibodies"[Mesh] OR "HIV Infections"[Mesh] OR "HIV Seroprevalence"[Mesh] OR HIV[Text] or "Human immunodeficiency virus"[Text]) |
| ***Women*** |
| "Female/analysis"[Mesh] OR "Female/statistics and numerical data"[Mesh] OR “Women/epidemiology”[Mesh] OR “Women/statistics and numerical data”[Mesh] OR Women[Text] OR Girl*[Text] OR Female*[Text] |
| **FINAL PUBMED SEARCH** |
| **(“Sex work” AND “Herpes simplex virus-2” AND “HIV” AND “Women”)** |
| **Total citations: 748** |
|  |
| **Embase** (September 3rd, 2019) |
| ***Sex work*** |
| exp prostitution/ or exp casual sex/ or exp transactional sex/ or exp group sex/ or exp sex tourism/ or exp sexual promiscuity/ or exp extramarital sex/ or exp premarital sex/ or exp sexual relation/ or exp sexual partners/ or ((exp sex trafficking/ or exp sexual exploitation/ or exp sexual coercion/) NOT Child) or (sex* work* or sexwork* or sex-work* or sex partner* or sexual partner* or sexual contact* or premarital sex or premarital sexual or premarital relation* or pre-marital sex or pre-marital sexual or pre-marital relation* or pre marital sex or pre marital sexual or pre marital relation* or extramarital sex or extramarital sexual or extramarital relation* or extra-marital sex or extra-marital sexual or extra-marital relation* or extra marital sex or extra marital sexual or extra marital relation* or illicit sex or illicit sexual or illicit relation* or illegal sex or illegal sexual or illegal relation* or (out* ADJ1 marriage) or illegal social behavio?r or adultery or prostitut* or promiscu* or FSW or FSWs or CSW or CSWs or SW or SWs or TSW or TSWs or TS or (women ADJ4 sex*) or (Travailleuse* ADJ1 sex*) or bar girl* or call girl* or callgirl* or escort* or masseuse* or hostess* or female entertain* or sex entertain* or sexual entertain* or entertainment work* or sex industr* or sex establishment* or brothel* or red light or red-light or (red ADJ1 district*) or nightclub* or pimp or recreation* sex* or intergenerational sex* or cross-generation sex* or cross-generational sex* or commercial sex* or transactional sex* or sex* transaction* or casual sex* or informal sex* or group sex* or street sex* or (migra* ADJ4 sex*) or (sex* ADJ4 migra*) or survival sex* or occupational sex* or sex* tourism or sex seeking or sex-seeking or solicit* or (consum* ADJ4 sex*) or (sex* ADJ 4 consumer) or (sex* ADJ4 consumers) or (sex* ADJ4 provi*) or (provi* ADJ4 sex*) or (sell* ADJ4 sex*) or (sex* ADJ4 sell*) or sold sex* or (exchang* ADJ4 sex*) or (sex* ADJ4 exchange) or (trading ADJ4 sex*) or (trade* ADJ4 sex*) or sex* trade or sex* favor* or (commodi* ADJ4 sex*) or (sex* ADJ4 commodi*) or (paid ADJ4 sex*) or (pay* ADJ4 sex*) or (sex* ADJ4 pay*) or (buy* ADJ4 sex*) or (sex* ADJ4 buy*) or (charg* ADJ4 sex*) or (sex* ADJ4 charg*) or (engag* ADJ4 sex*) or (sex* ADJ4 engage*) or (sex* ADJ4 service*) or (service* ADJ4 sex*) or (money ADJ4 sex*) or (sex* ADJ4 money) or (cash ADJ4 sex*) or (sex* ADJ4 cash) or (sex* ADJ4 drug*) or (drug* ADJ4 sex*) or (sex* ADJ4 goods) or (goods ADJ4 sex*) or (sex* ADJ4 gift*) or (gift* ADJ4 sex*) or hidden population* or hard to reach population* or hard-to-reach population* or (core ADJ1 group*) or vulnerable women or vulnerable female*).mp. or ((vulnerable population* or most-at-risk population* or most at risk population* or high risk population* or high-risk population* or population* at high risk or population* at high-risk).mp. AND (sex* or infection* or STI or STIs or STD or STDs or human immunodeficiency virus or HIV* or AIDS* or acquired immune deficiency syndrome or acquired immunodeficiency syndrome).mp.) or ((sex trafficking or sexual trafficking or (traffick* ADJ4 sex*) or sex* slave* or sex* coerc* or sex* abduct* or sex* exploit* or sex* abuse* or sex* violence) NOT Child).mp. or ((women ADJ4 traffick*) or (girls ADJ4 traffick*) or (female* ADJ4 traffick*) or (traffick* ADJ4 women) or (traffick* ADJ4 girls) or (traffick* ADJ4 female*)).mp. |
| ***Herpes simplex virus-2*** |
| (exp Herpes simplex virus/ or exp herpes simplex/ or exp Simplexvirus/ or exp Herpesvirus/ or exp Herpesviridae/ or exp Herpes simplex virus 2/) OR (Herpes simplex or Herpes simplex virus or HSV type-2 or HSV type 2 or HSV2 or HSV-2 or HSV 2 or HHV2 or HHV-2 or HHV 2 or human herpes virus or herpes virus or Herpes simplex virus type 2 or Herpes simplex virus type-2 or herpes simplex virus 2 or herpes simplex virus-2 or herpes simplex type 2 or herpes simplex type-2 or herpes simplex 2 or herpes simplex-2 or Herpesvirus type 2 or Herpesvirus type-2 or Herpesvirus 2 or Herpesvirus-2 or Herpes virus type 2 or Herpes virus type-2 or Herpes virus 2 or Herpes virus-2 or genital herpes or Herpes Genitalis or Herpes Labialis).mp. |
| ***HIV*** |
| (exp Human immunodeficiency virus/ or Human immunodeficiency virus.mp. or HIV.mp.) |
| ***Women*** |
| exp female/ or (women or girl* or female*).mp. |
|  |
| **FINAL EMBASE SEARCH** |
| **(“Sex work” AND “Herpes simplex virus-2” AND “HIV” AND “Women”)** |
| **Total citations: 1512** |
|  |
| **Abstract archives of the International AIDS Society conferences** (October 27, 2019) |
| “HIV” AND “HSV” |
| **Total citations: 63** |
| “HSV” |
| **Total citations: 496** |
| “Herpes” |
| **Total citations: 567** |

**Box S2.** List of extracted variables.

| **Report characteristics** |
| --- |
| Author(s) |
| Year of publication |
| Full citation |
| Publication type |
| Data source |
|  |
| **General study characteristics** |
| Study population and its characteristics |
| Year(s) of data collection |
| Region |
| Country of origin |
| Country of survey |
| City |
| Study site |
| Study design |
| Sampling methodology |
| Eligibility criteria |
|  |
| **HIV prevalence** |
| Number tested for HIV antibody |
| Number positive for HIV antibody |
| Reported HIV antibody prevalence |
| Diagnostic test used for HIV infection ascertainment |
|  |
| **Herpes simplex virus type 2 (HSV-2) prevalence** |
| Number tested for HSV-2 antibody |
| Number positive for HSV-2 antibody |
| Reported HSV-2 antibody prevalence |
| Diagnostic test used for HSV-2 infection ascertainment |
|  |
| **Population characteristics** |
| Proportion who inject drugs |
| Proportion on antiretroviral therapy |
| Proportion reporting consistent condom use |

**Box S3.** Countries covered under the different World Health Organization regions^80^.

| **World Health Organization region** | **Countries** |
| --- | --- |
| African Region (AFRO) | [Algeria](http://www.who.int/countries/dza/en/), [Angola](http://www.who.int/countries/ago/en/), [Benin](http://www.who.int/countries/ben/en/), [Botswana](http://www.who.int/countries/bwa/en/), [Burkina Faso](http://www.who.int/countries/bfa/en/), [Burundi](http://www.who.int/countries/bdi/en/), [Cameroon](http://www.who.int/countries/cmr/en/), [Cabo Verde](http://www.who.int/countries/cpv/en/), [Central African Republic](http://www.who.int/countries/caf/en/), [Chad](http://www.who.int/countries/tcd/en/), [Comoros](http://www.who.int/countries/com/en/), [Congo](http://www.who.int/countries/cog/en/), [Côte d'Ivoire](http://www.who.int/countries/civ/en/), [Democratic Republic of the Congo](http://www.who.int/countries/cod/en/), [Equatorial Guinea](http://www.who.int/countries/gnq/en/), [Eritrea](http://www.who.int/countries/eri/en/), [Ethiopia](http://www.who.int/countries/eth/en/), [Gabon](http://www.who.int/countries/gab/en/), [Gambia](http://www.who.int/countries/gmb/en/), [Ghana](http://www.who.int/countries/gha/en/), [Guinea](http://www.who.int/countries/gin/en/), [Guinea-Bissau](http://www.who.int/countries/gnb/en/), [Kenya](http://www.who.int/countries/ken/en/), [Lesotho](http://www.who.int/countries/lso/en/), [Liberia](http://www.who.int/countries/lbr/en/), [Madagascar](http://www.who.int/countries/mdg/en/), [Malawi](http://www.who.int/countries/mwi/en/), [Mali](http://www.who.int/countries/mli/en/), [Mauritania](http://www.who.int/countries/mrt/en/), [Mauritius](http://www.who.int/countries/mus/en/), [Mozambique](http://www.who.int/countries/moz/en/), [Namibia](http://www.who.int/countries/nam/en/), [Niger](http://www.who.int/countries/ner/en/), [Nigeria](http://www.who.int/countries/nga/en/), [Rwanda](http://www.who.int/countries/rwa/en/), [Sao Tome and Principe](http://www.who.int/countries/stp/en/), [Senegal](http://www.who.int/countries/sen/en/), [Seychelles](http://www.who.int/countries/syc/en/), [Sierra Leone](http://www.who.int/countries/sle/en/), [South Africa](http://www.who.int/countries/zaf/en/), [South Sudan](http://www.who.int/countries/ssd/en/), [Swaziland](http://www.who.int/countries/swz/en/), [Togo](http://www.who.int/countries/tgo/en/), [Uganda](http://www.who.int/countries/uga/en/), [United Republic of Tanzania](http://www.who.int/countries/tza/en/), [Zambia](http://www.who.int/countries/zmb/en/), [Zimbabwe](http://www.who.int/countries/zwe/en/). |
| Region of the Americas (AMRO) | [Antigua and Barbuda](http://www.who.int/countries/atg/en/), [Argentina](http://www.who.int/countries/arg/en/), [Bahamas](http://www.who.int/countries/bhs/en/), [Barbados](http://www.who.int/countries/brb/en/), [Belize](http://www.who.int/countries/blz/en/), [Bolivia (Plurinational State of)](http://www.who.int/countries/bol/en/), [Brazil](http://www.who.int/countries/bra/en/), [Canada](http://www.who.int/countries/can/en/), [Chile](http://www.who.int/countries/chl/en/), [Colombia](http://www.who.int/countries/col/en/), [Costa Rica](http://www.who.int/countries/cri/en/), [Cuba](http://www.who.int/countries/cub/en/), [Dominica](http://www.who.int/countries/dma/en/), [Dominican Republic](http://www.who.int/countries/dom/en/), [Ecuador](http://www.who.int/countries/ecu/en/), [El Salvador](http://www.who.int/countries/slv/en/), [Grenada](http://www.who.int/countries/grd/en/), [Guatemala](http://www.who.int/countries/gtm/en/), [Guyana](http://www.who.int/countries/guy/en/), [Haiti](http://www.who.int/countries/hti/en/), [Honduras](http://www.who.int/countries/hnd/en/), [Jamaica](http://www.who.int/countries/jam/en/), [Mexico](http://www.who.int/countries/mex/en/), [Nicaragua](http://www.who.int/countries/nic/en/), [Panama](http://www.who.int/countries/pan/en/), [Paraguay](http://www.who.int/countries/pry/en/), [Peru](http://www.who.int/countries/per/en/), [Saint Kitts and Nevis](http://www.who.int/countries/kna/en/), [Saint Lucia](http://www.who.int/countries/lca/en/), [Saint Vincent and the Grenadines](http://www.who.int/countries/vct/en/), [Suriname](http://www.who.int/countries/sur/en/), [Trinidad and Tobago](http://www.who.int/countries/tto/en/), [United States of America](http://www.who.int/countries/usa/en/), [Uruguay](http://www.who.int/countries/ury/en/), [Venezuela (Bolivarian Republic of)](http://www.who.int/countries/ven/en/). |
| Eastern Mediterranean Region (EMRO) | [Afghanistan](http://www.who.int/countries/afg/en/), [Bahrain](http://www.who.int/countries/bhr/en/), [Djibouti](http://www.who.int/countries/dji/en/), [Egypt](http://www.who.int/countries/egy/en/), [Iran (Islamic Republic of)](http://www.who.int/countries/irn/en/), [Iraq](http://www.who.int/countries/irq/en/), [Jordan](http://www.who.int/countries/jor/en/), [Kuwait](http://www.who.int/countries/kwt/en/), [Lebanon](http://www.who.int/countries/lbn/en/), [Libya](http://www.who.int/countries/lby/en/), [Morocco](http://www.who.int/countries/mar/en/), [Oman](http://www.who.int/countries/omn/en/), [Pakistan](http://www.who.int/countries/pak/en/), [Qatar](http://www.who.int/countries/qat/en/), [Saudi Arabia](http://www.who.int/countries/sau/en/), [Somalia](http://www.who.int/countries/som/en/), [Sudan](http://www.who.int/countries/sdn/en/), [Syrian Arab Republic](http://www.who.int/countries/syr/en/), [Tunisia](http://www.who.int/countries/tun/en/), [United Arab Emirates](http://www.who.int/countries/are/en/), [Yemen](http://www.who.int/countries/yem/en/). |
| European Region (EURO) | [Albania](http://www.who.int/countries/alb/en/), [Andorra](http://www.who.int/countries/and/en/), [Armenia](http://www.who.int/countries/arm/en/), [Austria](http://www.who.int/countries/aut/en/), [Azerbaijan](http://www.who.int/countries/aze/en/), [Belarus](http://www.who.int/countries/blr/en/), [Belgium](http://www.who.int/countries/bel/en/), [Bosnia and Herzegovina](http://www.who.int/countries/bih/en/), [Bulgaria](http://www.who.int/countries/bgr/en/), [Croatia](http://www.who.int/countries/hrv/en/), [Cyprus](http://www.who.int/countries/cyp/en/), [Czech Republic](http://www.who.int/countries/cze/en/), [Denmark](http://www.who.int/countries/dnk/en/), [Estonia](http://www.who.int/countries/est/en/), [Finland](http://www.who.int/countries/fin/en/), [France](http://www.who.int/countries/fra/en/), [Georgia](http://www.who.int/countries/geo/en/), [Germany](http://www.who.int/countries/deu/en/), [Greece](http://www.who.int/countries/grc/en/), [Hungary](http://www.who.int/countries/hun/en/), [Iceland](http://www.who.int/countries/isl/en/), [Ireland](http://www.who.int/countries/irl/en/), [Israel](http://www.who.int/countries/isr/en/), [Italy](http://www.who.int/countries/ita/en/), [Kazakhstan](http://www.who.int/countries/kaz/en/), [Kyrgyzstan](http://www.who.int/countries/kgz/en/), [Latvia](http://www.who.int/countries/lva/en/), [Lithuania](http://www.who.int/countries/ltu/en/), [Luxembourg](http://www.who.int/countries/lux/en/), [Malta](http://www.who.int/countries/mlt/en/), [Monaco](http://www.who.int/countries/mco/en/), [Montenegro](http://www.who.int/countries/mnt/en/), [Netherlands](http://www.who.int/countries/nld/en/), [Norway](http://www.who.int/countries/nor/en/), [Poland](http://www.who.int/countries/pol/en/), [Portugal](http://www.who.int/countries/prt/en/), [Republic of Moldova](http://www.who.int/countries/mda/en/), [Romania](http://www.who.int/countries/rou/en/), [Russian Federation](http://www.who.int/countries/rus/en/), [San Marino](http://www.who.int/countries/smr/en/), [Serbia,](http://www.who.int/countries/scg/en/) [Slovakia](http://www.who.int/countries/svk/en/), [Slovenia](http://www.who.int/countries/svn/en/), [Spain](http://www.who.int/countries/esp/en/), [Sweden](http://www.who.int/countries/swe/en/), [Switzerland](http://www.who.int/countries/che/en/), [Tajikistan](http://www.who.int/countries/tjk/en/), [The former Yugoslav Republic of Macedonia](http://www.who.int/countries/mkd/en/), [Turkey](http://www.who.int/countries/tur/en/), [Turkmenistan](http://www.who.int/countries/tkm/en/), [Ukraine](http://www.who.int/countries/ukr/en/), [United Kingdom](http://www.who.int/countries/gbr/en/), [Uzbekistan](http://www.who.int/countries/uzb/en/). |
| South-East Asia Region (SEARO) | [Bangladesh](http://www.who.int/countries/bgd/en/), [Bhutan](http://www.who.int/countries/btn/en/), [Democratic People's Republic of Korea](http://www.who.int/countries/prk/en/), [India](http://www.who.int/countries/ind/en/), [Indonesia](http://www.who.int/countries/idn/en/), [Maldives](http://www.who.int/countries/mdv/en/), [Myanmar](http://www.who.int/countries/mmr/en/), [Nepal](http://www.who.int/countries/npl/en/), [Sri Lanka](http://www.who.int/countries/lka/en/), [Thailand](http://www.who.int/countries/tha/en/), [Timor-Leste](http://www.who.int/countries/tls/en/). |
| Western Pacific Region (WPRO) | [Australia](http://www.who.int/countries/aus/en/), [Brunei Darussalam](http://www.who.int/countries/brn/en/), [Cambodia](http://www.who.int/countries/khm/en/), [China](http://www.who.int/countries/chn/en/), [Cook Islands](http://www.who.int/countries/cok/en/), [Fiji](http://www.who.int/countries/fji/en/), [Japan](http://www.who.int/countries/jpn/en/), [Kiribati](http://www.who.int/countries/kir/en/), [Lao People's Democratic Republic](http://www.who.int/countries/lao/en/), [Malaysia](http://www.who.int/countries/mys/en/), [Marshall Islands](http://www.who.int/countries/mhl/en/), [Micronesia (Federated States of)](http://www.who.int/countries/fsm/en/), [Mongolia](http://www.who.int/countries/mng/en/), [Nauru](http://www.who.int/countries/nru/en/), [New Zealand](http://www.who.int/countries/nzl/en/), [Niue](http://www.who.int/countries/niu/en/), [Palau](http://www.who.int/countries/plw/en/), [Papua New Guinea](http://www.who.int/countries/png/en/), [Philippines](http://www.who.int/countries/phl/en/), [Republic of Korea](http://www.who.int/countries/kor/en/), [Samoa](http://www.who.int/countries/wsm/en/), [Singapore](http://www.who.int/countries/sgp/en/), [Solomon Islands](http://www.who.int/countries/slb/en/), [Tonga](http://www.who.int/countries/ton/en/), [Tuvalu](http://www.who.int/countries/tuv/en/), [Vanuatu](http://www.who.int/countries/vut/en/), [Viet Nam](http://www.who.int/countries/vnm/en/). |

**References**

1. Low, A. J. *et al.* Genital warts and infection with human immunodeficiency virus in high-risk women in Burkina Faso: a longitudinal study. *BMC Infect. Dis.* **11**, 20-29; 10.1186/1471-2334-11-20 (2011).

2. Nagot, N. *et al.* *Prevalence, incidence and risk factors of HSV2 infection among a high-risk popilation in West Africa. Poster 999* in *International AIDS Conference* (2003).

3. Nagot, N. *et al.* Association between bacterial vaginosis and Herpes simplex virus type-2 infection: Implications for HIV acquisition studies. *Sex. Transm. Infect.* **83**, 365-368; 10.1136/sti.2007.024794 (2007).

4. Nzila, N. *et al.* HIV and other sexually transmitted diseases among female prostitutes in Kinshasa. *Aids* **5**, 715-721 (1991).

5. Vandepitte, J. M. *et al.* HIV and other sexually transmitted infections among female sex workers in Kinshasa, Democratic Republic of Congo, in 2002. *Sex Transm Dis* **34**, 203-208; 10.1097/01.olq.0000233743.57334.6a (2007).

6. Ghebrekidan, H., Ruden, U., Cox, S., Wahren, B. & Grandien, M. Prevalence of herpes simplex virus types 1 and 2, cytomegalovirus, and varicella-zoster virus infections in Eritrea. *J. Clin. Virol.* **12**, 53-64 (1999).

7. Holt, B. Y. *et al.* Planning STI/HIV prevention among refugees and mobile populations: situation assessment of Sudanese refugees. *Disasters* **27**, 1-15 (2003).

8. Aho, J., Koushik, A., Coutlee, F., Diakite, S. L. & Rashed, S. Prevalence of HIV, human papillomavirus type 16 and herpes simplex virus type 2 among female sex workers in Guinea and associated factors. *Int. J. STD AIDS* **25**, 280-288; 10.1177/0956462413500242 (2014).

9. Diakite, S. *et al.* *HIV transmission in a cohort of sex workers in Conakry, Guinea: HSV-2 baseline prevalence, characteristics of and its associations with HIV, other STIs and behavioural variables. CDC0197* in *International AIDS Conference* (2006).

10. Vandenhoudt, H. M. *et al.* Prevalence of HIV and Other Sexually Transmitted Infections among Female Sex Workers in Kisumu, Western Kenya, 1997 and 2008. *PLoS ONE* **8**; 10.1371/journal.pone.0054953 (2013).

11. Lafort Y., Sambola F., Joaquim F. & Temmerman M. *Low prevalence rates of STI among high-risk groups benefiting from improved reproductive health services in Tete province, Mozambique. Poster THPE0308* in *International AIDS Conference* (2008).

12. Dada, A. J. *et al.* A serosurvey of Haemophilus ducreyi, syphilis, and herpes simplex virus type 2 and their association with human immunodeficiency virus among female sex workers in Lagos, Nigeria. *Sex. Transm. Dis.* **25**, 237-242 (1998).

13. Eltom, M. A., Mbulaiteye, S. M., Dada, A. J., Whitby, D. & Biggar, R. J. Transmission of human herpesvirus 8 by sexual activity among adults in Lagos, Nigeria. *Aids* **16**, 2473-2478 (2002).

14. Braunstein, S. L. *et al.* High burden of prevalent and recently acquired HIV among female sex workers and female HIV voluntary testing center clients in Kigali, Rwanda. *PLoS ONE* **6**; 10.1371/journal.pone.0024321 (2011).

15. Kane, C. T. *et al.* Concentrated and linked epidemics of both HSV-2 and HIV-1/HIV-2 infections in Senegal: public health impacts of the spread of HIV. *Int. J. STD AIDS* **20**, 793-796; 10.1258/ijsa.2008.008414 (2009).

16. Malope, B. I. *et al.* No evidence of sexual transmission of Kaposi's sarcoma herpes virus in a heterosexual South African population. *Aids* **22**, 519-526; 10.1097/QAD.0b013e3282f46582 (2008).

17. Ramjee, G. *et al.* The impact of incident and prevalent herpes simplex virus-2 infection on the incidence of HIV-1 infection among commercial sex workers in South Africa. *J. Acquir. Immune Defic. Syndr.* **39**, 333-339 (2005).

18. Riedner, G. *et al.* Possible reasons for an increase in the proportion of genital ulcers due to herpes simplex virus from a cohort of female bar workers in Tanzania. *Sex. Transm. Infect.* **83**, 91-96; 10.1136/sti.2006.021287 (2007).

19. Vu, L. & Misra, K. High Burden of HIV, Syphilis and HSV-2 and Factors Associated with HIV Infection Among Female Sex Workers in Tanzania: Implications for Early Treatment of HIV and Pre-exposure Prophylaxis (PrEP). *AIDS Behav.* **22**, 1113-1121; 10.1007/s10461-017-1992-2 (2018).

20. Vandepitte, J. *et al.* HIV and other sexually transmitted infections in a cohort of women involved in high-risk sexual behavior in Kampala, Uganda. *Sex. Transm. Dis.* **38**, 316-323 (2011).

21. Cowan, F. M. *et al.* The appropriateness of core group interventions using presumptive periodic treatment among rural Zimbabwean women who exchange sex for gifts or money. *J. Acquir. Immune Defic. Syndr.* **38**, 202-207 (2005).

22. Alvarez Rodriguez, B., Manzanero, M. & Morales Miranda, S. Results of the first HIV prevalence and risk behaviour study among female sex workers, Belize, 2012. *Sex. Transm. Infect.* **89**; 10.1136/sextrans-2013-051184.0566 (2013).

23. Koenig, E. *et al.* *Characterization of a cohort of female sex workers in the Dominican Republic at risk for HIV and other STIs: implications for inclusion in future HIV-1 vaccine efficacy trials. Poster TUPDC04* in *4th IAS Conference on HIV Pathogenesis, Treatment and Prevention.* (2007).

24. Creswell, J., Guardado Escobar, M. E., Armero, J. & Paz-Bailey, G. *HIV, STD and risk behaviors among female sex worker in El Salvador. Poster CDC0622* in *International AIDS Conference* (2010).

25. Soto, R. J. *et al.* Sentinel surveillance of sexually transmitted infections/HIV and risk behaviors in vulnerable populations in 5 Central American countries. *J. Acquir. Immune Defic. Syndr.* **46**, 101-111 (2007).

26. Morales-Miranda, S. *et al.* *HIV, STD and risk behaviors among men who have sex with men, female sex workers, and indigenous Garífuna population in Honduras. WEAX0305* in *International AIDS Conference* (2008).

27. Uribe-Salas, F., Hernandez-Avila, M., Juarez-Figueroa, L., Conde-Glez, C. J. & Uribe-Zuniga, P. Risk factors for herpes simplex virus type 2 infection among female commercial sex workers in Mexico City. *Int. J. STD AIDS* **10**, 105-111 (1999).

28. Uribe-Salas, F., Conde-Glez, C. J., Juarez-Figueroa, L. & Hernandez-Castellanos, A. Sociodemographic dynamics and sexually transmitted infections in female sex workers at the Mexican-Guatemalan border. *Sex. Transm. Dis.* **30**, 266-271 (2003).

29. Delgado, S. *et al.* Central american surveillance survey of sexual behaviour and prevalence of HIV/STIS in vulnerable populations: Female sex workers, Nicaragua, 2009. *Sex. Transm. Infect.* **1**; 10.1136/sextrans-2011-050109.46 (2011).

30. Hakre, S. *et al.* Prevalence of HIV and other sexually transmitted infections and factors associated with syphilis among female sex workers in Panama. *Sex. Transm. Infect.* **89**, 156-164; 10.1136/sextrans-2012-050557 (2013).

31. Caceres, C. F. *et al.* *High risk for STIs among vulnerable populations in the Peruvian coast: the NIMH HIV/STI collaborative prevention trial. Poster TUPE0293* in *International AIDS Conference* (2006).

32. Carcamo, C. P. *et al.* Prevalences of sexually transmitted infections in young adults and female sex workers in Peru: a national population-based survey. *Lancet Infect. Dis.* **12**, 765-773 (2012).

33. Golenbock, D. T. *et al.* Absence of infection with human immunodeficiency virus in Peruvian prostitutes. *AIDS Research and Human Retroviruses* **4**, 493-499 (1988).

34. Gotuzzo, E. *et al.* Human T cell lymphotropic virus type I infection among female sex workers in Peru. *J. Infect. Dis.* **169**, 754-759 (1994).

35. Perla, M. E. *et al.* Genital tract infections, bacterial vaginosis, HIV, and reproductive health issues among Lima-based clandestine female sex workers. *Infect. Dis. Obstet. Gynecol.* **2012**, 739624; 10.1155/2012/739624 (2012).

36. Sanchez, J. *et al.* Sexually transmitted infections in female sex workers: reduced by condom use but not by a limited periodic examination program. *Sex. Transm. Dis.* **25**, 82-89; 10.1097/00007435-199802000-00005 (1998).

37. Cohan, D. L. *et al.* Health indicators among low income women who report a history of sex work: the population based Northern California Young Women's Survey. *Sex. Transm. Infect.* **81**, 428-433; 10.1136/sti.2004.013482 (2005).

38. Jones, D. L. *et al.* The high-risk sexual practices of crack-smoking sex workers recruited from the streets of three American cities. *Sex. Transm. Dis.* **25**, 187-193 (1998).

39. Lutnick A., C. D. *Working conditions, HIV, STIs and hepatitis C among female sex workers in San Francisco, CA. Poster WEPE0773* in *International ADIS Conference* (2008).

40. Papadogeorgaki, H. *et al.* Prevalence of sexually transmitted infections in female sex workers in Athens, Greece - 2005. *Eur. J. Dermatol.* **16**, 662-665 (2006).

41. Linhart, Y. *et al.* Sexually transmitted infections among brothel-based sex workers in Tel-Aviv area, Israel: high prevalence of pharyngeal gonorrhoea. *Int. J. STD AIDS* **19**, 656-659; 10.1258/ijsa.2008.008127 (2008).

42. Khromova, Y. Y. *et al.* *High rates of sexually transmitted diseases (STDs), HIV and risky behaviors among female detainees in Moscow, Russia. Poster ThPeC7600* in *International AIDS Conference* (2002).

43. Bystricka, M. *et al.* Sexually transmitted infections among prostitutes in Bratislava, Slovakia. *Acta Virol.* **47**, 167-172 (2003).

44. Gul, U. *et al.* Magnitude of sexually transmitted infections among female sex workers in Turkey. *J. Eur. Acad. Dermatol. Venereol.* **22**, 1123-1124 (2008).

45. Hawkes, S. *et al.* HIV and other sexually transmitted infections among men, transgenders and women selling sex in two cities in Pakistan: a cross-sectional prevalence survey. *Sex. Transm. Infect.* **85**, ii8-16 (2009).

46. Znazen, A. *et al.* Sexually transmitted infections among female sex workers in Tunisia: high prevalence of Chlamydia trachomatis. *Sex. Transm. Infect.* **86**, 500-505 (2010).

47. Qutub, M. & Akhter, J. Epidemiology of genital herpes (HSV-2) among brothel based female sex workers in Bangladesh. *Eur. J. Epidemiol.* **18**, 903-905 (2003).

48. Pisani, E. *et al.* Basing policy on evidence: Low HIV, STIs, and risk behaviour in Dili, East Timor argue for more focused interventions. *Sex. Transm. Infect.* **82**, 88-93; 10.1136/sti.2005.015602 (2006).

49. Mishra, S. *et al.* Sex work, syphilis, and seeking treatment: an opportunity for intervention in HIV prevention programming in Karnataka, South India. *Sex. Transm. Dis.* **36**, 157-164; 10.1097/OLQ.0b013e31818d64e6 (2009).

50. National Summary Report - India. *Integrated Behavioural and Biological Assessment, Round 2 (2009-2010)*. (2011).

51. Sarna, A. *et al.* Sexually transmitted infections and reproductive health morbidity in a cohort of female sex workers screened for a microbicide feasibility study in Nellore, India. *Glob. J. Health. Sci.* **5**, 139-149; 10.5539/gjhs.v5n3p139 (2013).

52. Shahmanesh, M. *et al.* The burden and determinants of HIV and sexually transmitted infections in a population-based sample of female sex workers in Goa, India. *Sex. Transm. Infect.* **85**, 50-59; 10.1136/sti.2008.030767 (2009).

53. Uma, S. *et al.* Bacterial vaginosis in female sex workers in Chennai, India. *Sex. Health* **2**, 261-262; 10.1071/SH05025 (2005).

54. Davies, S. C. *et al.* Prevalence and risk factors for herpes simplex virus type 2 antibodies among low- and high-risk populations in Indonesia. *Sex. Transm. Dis.* **34**, 132-138 (2007).

55. Limpakarnjanarat, K. *et al.* HIV-1 and other sexually transmitted infections in a cohort of female sex workers in Chiang Rai, Thailand. *Sex. Transm. Infect.* **75**, 30-35 (1999).

56. Vu Thuong, N. *et al.* Impact of a community sexually transmitted infection/HIV intervention project on female sex workers in five border provinces of Vietnam. *Sex. Transm. Infect.* **83**, 376-382 (2007).

57. Saphonn, V. *et al.* *HSV-2 infection among female sex workers in Sihanoukville, Cambodia: high prevalence and strong association with HIV infection. Poster TUPE0294* in *International AIDS Conference* (2006).

58. Chen, Y. M., Yu, P. S., Lin, C. C. & Jen, I. Surveys of HIV-1, HTLV-I, and other sexually transmitted diseases in female sex workers in Taipei City, Taiwan, from 1993 to 1996. *J. Acquir. Immune Defic. Syndr. Hum. Retrovirol.* **18**, 299-303 (1998).

59. Chen, X. S. *et al.* Sexually transmitted infections among female sex workers in Yunnan, China. *AIDS Patient Care STDS* **19**, 853-860; 10.1089/apc.2005.19.853 (2005).

60. Chen, S. *et al.* Seropositivity and risk factors for herpes simplex virus type 2 infection among female sex workers in Guangxi, China. *PLoS ONE* **8** 10.1371/journal.pone.0069697 (2013).

61. Fu, X. *et al.* Prevalence of HIV and sexually transmitted diseases as well as related associated risk factors among middle/low level female sex workers in a city in Guangdong province. [Chinese]. *Zhonghua Liu Xing Bing Xue Za Zhi* **35**, 510-513 (2014).

62. Han, L. *et al.* Differences in risk behaviours and HIV/STI prevalence between low-fee and medium-fee female sex workers in three provinces in China. *Sex. Transm. Infect.* **92**, 309-315 (2016).

63. Jing, Z. *et al.* Consecutive cross-sectional survey of prevalence of HIV infection/STD and related factors in Vietnamese female sex workers at a China-Vietnam border area, 2014-2015. [Chinese]. *Chinese Journal of Endemiology* **38**, 638-642 (2017).

64. Li, Z. *et al.* Study on the prevalence and associated risk factors related to HIV, syphilis, herpes simplex virus-2 among female sex workers in Jiaozhou, Shandong province. [Chinese]. *Zhonghua Liu Xing Bing Xue Za Zhi* **35**, 1099-1104 (2014).

65. Luo, L. *et al.* Vaginal douching and association with sexually transmitted infections among female sex workers in a prefecture of Yunnan Province, China. *Int. J. STD AIDS* **27**, 560-567 (2015).

66. Ngo, T. D. *et al.* Herpes simplex virus type 2 infection among commercial sex workers in Kunming, Yunnan Province, China. *Int. J. STD AIDS* **19**, 694-697 (2008).

67. Remis, R. S. *et al.* *Prevalence of HIV infection and STI among female entertainment workers (FEWs) in Shanghai, China. Poster TUPE0361* in *International AIDS Conference* (2010).

68. Wang N., Y. Z., Gao H., Duan Q., Zhao R., Lu L., Pu Y., Ni W., Wu Z.,. *HIV infection and other sexually transmitted infections among female sex workers in a mining township in Yunnan, China. Poster TUPE0297* in *International AIDS Conference* (2006).

69. Wang, J. J. *et al.* Estimation of population-size changes and HIV prevalence among female sex workers from 2006 to 2009 in Kaiyuan, Yunnan, China. *Biomed. Environ. Sci.* **25**, 489-494 (2012).

70. Wang, J., Ding, G., Zhu, Z., Zhou, C. & Wang, N. Analysis of HIV correlated factors in Chinese and Vietnamese female sex workers in Hekou, Yunnan Province, a Chinese Border Region. *PLoS ONE* **10**; 10.1371/journal.pone.0129430 (2015).

71. Wei, S. B. *et al.* A study of commercial sex and HIV/STI-related risk factors among hospitality girls in entertainment establishments in Wuhan, China. *Sex. Health* **1**, 141-144 (2004).

72. Xu, J. J. *et al.* HIV and STIs in clients and female sex workers in mining regions of Gejiu City, China. *Sex. Transm. Dis.* **35**, 558-565 (2008).

73. Xu, J. J. *et al.* Dynamics of the HIV epidemic in southern China: sexual and drug-using behaviours among female sex workers and male clients in Yunnan. *Int. J. STD AIDS* **23**, 670-675 (2012).

74. Xu, J. *et al.* Drug use and sex work: Competing risk factors for newly acquired HIV in Yunnan, China. *PLoS ONE* **8**; 10.1371/journal.pone.0059050 (2013).

75. Yang, Y. *et al.* Herpes simplex virus type 2 infection among female sex workers in Shanghai, China. *AIDS Care* **23**, 37-44 (2011).

76. Yao, Y. *et al.* Associations between drug use and risk behaviours for HIV and sexually transmitted infections among female sex workers in Yunnan, China. *Int. J. STD AIDS* **23**, 698-703 (2012).

77. Zhang, T. *et al.* Kaposi`s sarcoma associated herpesvirus infection among female sex workers and general population women in Shanghai, China: a cross-sectional study. *BMC Infect. Dis.* **14**; 10.1186/1471-2334-14-58 (2014).

78. Tableau. Tableau v. 10.1. Available at: <https://www.tableau.com/support/releases/desktop/10.1>. (2016).

79. R Core Team. *R: A language and environment for statistical computing. v.3.4.2*. (R Foundation for Statistical Computing, 2017).

80. World Health Organization. *WHO regional offices.* Available at: <http://www.who.int/about/regions/en/>. (2020).
